# Haematopoietic stem and progenitor cells from human pluripotent stem cells

**DOI:** 10.1038/nature22370

**Published:** 2017-05-17

**Authors:** Ryohichi Sugimura, Deepak Kumar Jha, Areum Han, Clara Soria-Valles, Edroaldo Lummertz da Rocha, Yi-Fen Lu, Jeremy A. Goettel, Erik Serrao, R. Grant Rowe, Mohan Malleshaiah, Irene Wong, Patricia Sousa, Ted N. Zhu, Andrea Ditadi, Gordon Keller, Alan N. Engelman, Scott B. Snapper, Sergei Doulatov, George Q. Daley

**Affiliations:** 1 Stem Cell Transplantation Program, Division of Pediatric Hematology and Oncology, Dana-Farber Cancer Institute, Boston Children's Hospital and Dana-Farber Cancer Institute, Boston, Massachusetts 02115, USA; 2 Department of Biological Chemistry and Molecular Pharmacology, Harvard Medical School, Boston, Massachusetts 02115, USA; 3 Harvard Stem Cell Institute, Cambridge, Massachusetts 02138, USA; 4 Manton Center for Orphan Disease Research, Boston, Massachusetts 02115, USA; 5 Department of Pediatrics, Division of Gastroenterology, Hepatology and Nutrition, Boston Children's Hospital, Boston, Massachusetts, USA; 6 Department of Medicine, Harvard Medical School, Boston, Massachusetts, USA; 7 Department of Cancer Immunology and Virology, Dana-Farber Cancer Institute and Department of Medicine, Harvard Medical School, Boston, Massachusetts, 02215, USA; 8 Department of Systems Biology, Harvard Medical School, Boston, Massachusetts, USA; 9 Department of Biology, Brandeis University, Waltham, Massachusetts 02453, USA; 10 Program in Computer Science, Harvard University, Cambridge, Massachusetts, USA; 11 McEwen Centre for Regenerative Medicine, University Health Network, Toronto, Ontario M5G 1L7, Canada; 12 Division of Gastroenterology, Brigham and Women's Hospital, Boston, Massachusetts, USA; 13 Howard Hughes Medical Institute, Boston, Massachusetts 02115, USA

## Abstract

A variety of tissue lineages can be differentiated from pluripotent stem cells by mimicking embryonic development through stepwise exposure to morphogens, or by conversion of one differentiated cell type into another by enforced expression of master transcription factors. Here, to yield functional human haematopoietic stem cells, we perform morphogen-directed differentiation of human pluripotent stem cells into haemogenic endothelium followed by screening of 26 candidate haematopoietic stem-cell-specifying transcription factors for their capacity to promote multi-lineage haematopoietic engraftment in mouse hosts. We recover seven transcription factors (*ERG*, *HOXA5*, *HOXA9*, *HOXA10*, *LCOR*, *RUNX1* and *SPI1*) that are sufficient to convert haemogenic endothelium into haematopoietic stem and progenitor cells that engraft myeloid, B and T cells in primary and secondary mouse recipients. Our combined approach of morphogen-driven differentiation and transcription-factor-mediated cell fate conversion produces haematopoietic stem and progenitor cells from pluripotent stem cells and holds promise for modelling haematopoietic disease in humanized mice and for therapeutic strategies in genetic blood disorders.

Cell identity is defined by gene regulatory networks that are governed by transcription factors^1^. By supplying transcription factors that drive haematopoietic gene regulatory networks, several groups have gene rated haematopoietic cells from sources as diverse as fibroblasts, endothelial cells, and differentiated blood cells^2–7^. In a previous study, we screened nine haematopoietic stem cell (HSC)-specific transcription factors for their potential to induce *in vitro* haematopoietic colony-forming activity and *in vivo* engraftment from human pluripotent stem cell (hPSC)-derived myeloid cells, and isolated five transcription factors (*HOXA9*, *ERG*, *RORA*, *SOX4*, and *MYB*) that promoted short-term engraftment of erythroid and myeloid cells, but did not achieve long-term multi-lineage haematopoiesis^7^. Building upon evidence that HSCs are derived from definitive haemogenic endothelium^8–12^, recent approaches have recapitulated haemogenic endothelium differentiation from hPSCs to generate cells with myeloid and T-cell haematopoietic potential *in vitro*^13–15^; however, few, if any, cells capable of engrafting irradiated mouse hosts were generated.

## *In vivo* screening of transcription factors

**Extended Data Figure 1:**
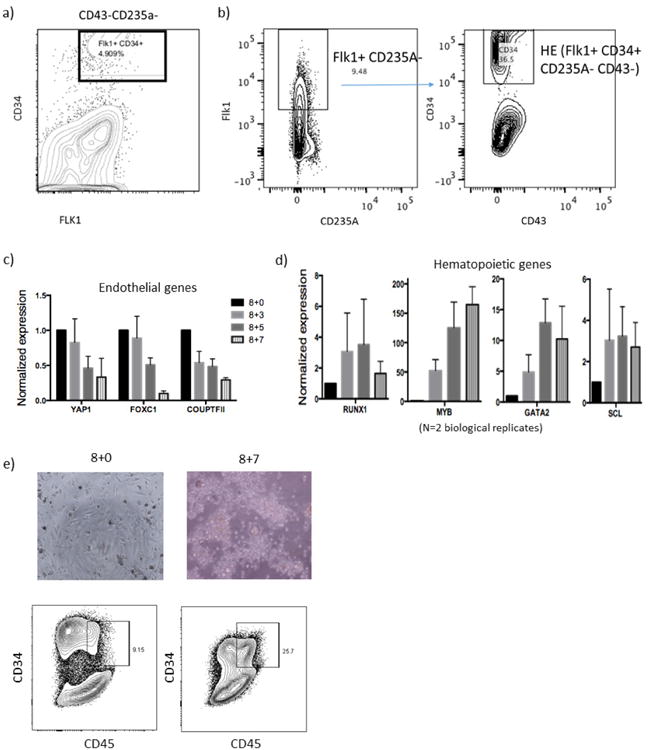
Induction of haemogenic endothelium from hPSCs **a**, Embryoid bodies formed from hPSCs. Subsequent panels show FACS analysis of day 8 embryoid bodies before magnetic cell isolation. **b**, CD34^+^FLK1^+^ population indicates haemogenic endothelium cells.CD235A and FLK1 plots show percentage of FLK1^+^CD235A^−^ cells in embryoid bodies, further gated with CD43 and CD34 plots to detect haemogenic endothelium (CD34^+^FLK1^+^CD235A^−^CD43^−^). **c, d**, Haemogenic endothelium isolated at day 8 (Supplementary Fig. 1) was further cultured in EHT medium for the indicated number of days. qRT–PCR showed (**c**) downregulation of endothelial genes and (**d**) upregulation of haematopoietic genes. HSPC genes (*RUNX1*, *SCL*/*TAL1*) peak on day 3 of EHT culture; consequently, this time point was chosen for introducing transcription factors followed by transplantation in subsequent experiments. **e**, Microscopy and FACS analysis on day 7 of EHT showed the appearance of haematopoietic cells (CD34^+^CD45^+^). Data shown as mean ± s.d.

We adapted a protocol to derive haemogenic endothelium from hPSCs and verified haematopoietic potential^14^. We isolated haemogenic endothelium on the basis of magnetic cell isolation of a CD34^+^ population, which enriched for FLK1^+^CD43^−^CD235A^−^ cells at day 8 of embryoid body formation (Extended Data Fig. 1a, b). Upon further culture with haematopoietic cytokines, we observed an endothelial-to-haematopoietic transition (EHT). Consistent with previous reports^13,14^, we documented a decrease in expression of endothelial genes (*YAP, FOXC1, COUPTFII*), an increase in levels of haematopoietic lineage genes (*RUNX1, MYB, GATA2, SCL*), and concomitant emergence of CD34^+^CD45^+^ haematopoietic cells (Extended Data Fig. 1c–e). However, multiple attempts to engraft irradiated immune-deficient recipient mice with these cultured cells failed.

**Extended Data Figure 2:**
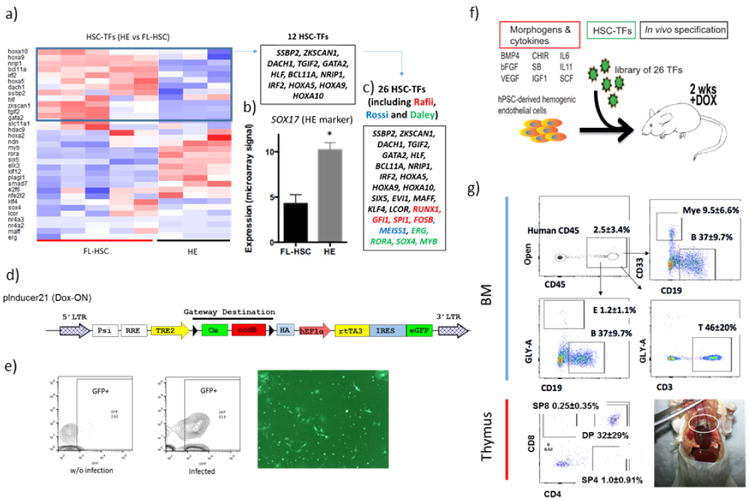
Rationale for selecting candidate transcription factors for library screening **a**, Heatmap of the expression profile of HSC-specific transcription factors (TFs) in haemogenic endothelium (CD34^+^FLK1^+^CD43^−^CD235A^−^) versus fetal-liver HSCs (CD34^+^CD38^−^CD90^+^CD45^+^). Twelve HSC-specific transcription factors enriched in fetal-liver HSCs relative to haemogenic endothelium (blue box) were cloned individually into a Dox-inducible lentiviral vector. **b**, The expression level of *SOX17*, a marker of haemogenic endothelium, was 2.4-fold higher in haemogenic endothelium (*N* = 7) than fetal-liver HSCs (*N* = 10). *P < 0.001. **c**, The library was supplemented with genes identified in previous screens. Candidates in red were drawn from a previous screen by the Rafii group^3^; blue from the Rossi group^2^; green from the Daley group^7^. The final library of 26 candidates is shown. **d**, Diagram of the Dox-ON pInducer-21 lentiviral vector used in this study (top). rtTA3 and eGFP are driven by EF1α -promoter; infection efficiency is indicated by the GFP signal. **e**, GFP analysis by FACS and fluorescence 3 days after infection of haemogenic endothelium cells. Routinely, over 50% transduction efficiency was achieved. For transplantation, haemogenic endothelium cells infected at day 3 EHT were incubated for 24 h and injected into mice. Dox was provided for 2 weeks *in vivo* after transplantation into sub-lethally irradiated immune-deficient NSG mice. **f**, Scheme for screening the 26 transcription factors library, and resulting haematopoietic chimaerism. hPSC-derived haemogenic endothelium was cultured for an additional 3 days in EHT medium, then infected with the library of 26 transcription factors. Infected cells (100,000) were injected intrafemorally into sub-lethally irradiated (250 rad) NSG mice, which were treated with doxycycline for 2 weeks to induce transgene expression *in vivo*. **g**, FACS analysis of bone marrow and thymus of an engrafted recipient is shown. Human CD45^+^ cells from bone marrow were analysed for CD33^+^ myeloid cells, CD19^+^ B cells, and CD3^+^ T cells as indicated. Thymic cells were analysed for human CD4 and CD8, with percentages of single and double-positive cells indicated. A photograph of an engrafted thymus is shown (bottom right). Data shown as mean ± s.d.

**Extended Data Figure 3:**
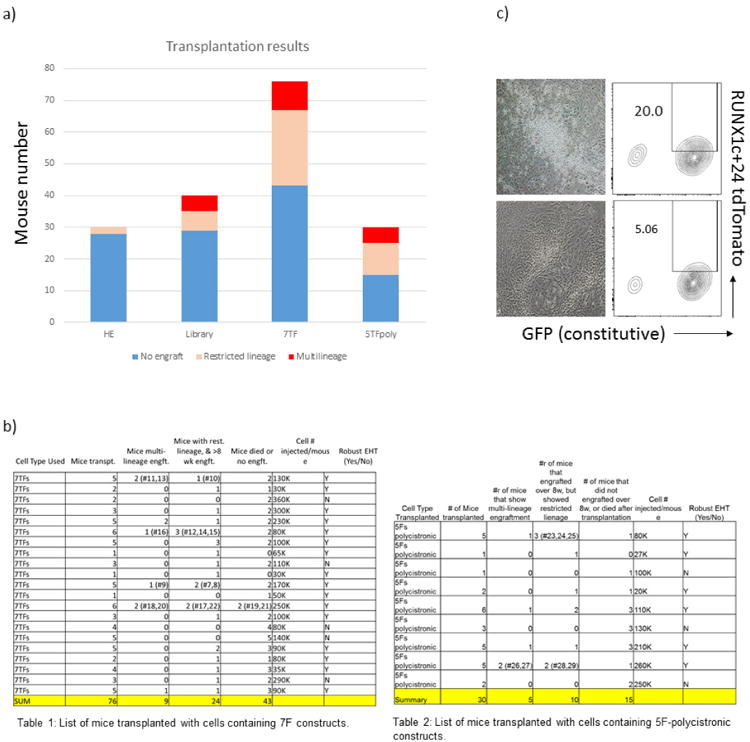
Compiled data from transplantation experiments performed so far **a, b**, Total engrafted mice assessed in primary transplantation at various time points. **a**, Histogram of total mice injected with haemogenic endothelium cells and haemogenic endothelium cells infected with indicated library or constituent transcription factors. Haemogenic endothelium only: 2 out of 30 mice engrafted, none of which were multi-lineage (myeloid cells, erythroid cells, B cells and T cells); library: 11 out of 40 mice engrafted, 5 multi-lineage; 7 transcription factors: 33 out of 76 mice engrafted, 9 multi-lineage; 5TFpoly: 15 out of 30 mice engrafted, 5 multi-lineage. Engraftment was assessed from bone marrow at 4–16 weeks. **b**, Tables 1 and 2 show compiled data. **c**, Representative photomicrographs and FACS plots of robust EHT (top) and failed EHT (bottom). The appearance of round cells budding from adherent haemogenic endothelium cells by direct microscopic visualization was assessed routinely. RUNX1c+24 reporter positivity of haemogenic endothelium cells at day 3 of culture likewise reflected robustness of EHT (20% versus 5%).

**Extended Data Figure 4:**
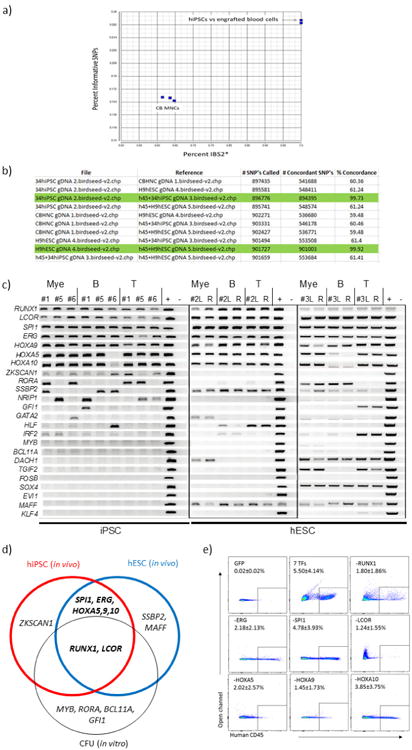
Identification of transcription factors that confer multi-lineage haematopoiesis *in vivo* **a**, SNP analysis of engrafted blood cells compared with hiPSC and cord blood MNCs. SNP array genotyping was conducted to confirm the origin of engrafted human cells from hiPSCs in representative mice. SNP genotypes for human CD45^+^ cells taken from bone marrow of engrafted mice, original hPSCs and reference cord blood MNCs were clustered, as shown, showing concordance of original hiPSCs and human cells recovered from bone marrow of engrafted mice. **b**, Tabular presentation of SNP data: concordance > 99% indicates identity between cell types. Green highlight shows that original hPSC line (34hiPSC or H9 hESC) corresponds with human CD45^+^ cells in engrafted mice. Comparison with different hPSC lines or cord blood MNCs did not achieve 99% concordance, validating the SNP array as a means of defining origin of cells. **c**, Transgene detection in engrafted cells of primary recipients. CD33^+^ myeloid cells, CD19^+^ B cells, and CD3^+^ T cells were isolated from the human CD45^+^ population of bone marrow at 10 weeks from five independent mice. Genomic DNA of each cell type was analysed by PCR to detect integrated lentivirus. Identification number of recipients is shown (numbers 1, 5, and 6 were engrafted with hiPSC-derived haemogenic endothelium; numbers 2 and 3 were engrafted with hESC-derived haemogenic endothelium). L, left femur (injected side); R, right femur; +, positive control (lentiviral vector with each transcription factor). –, negative control (lentiviral vector without transcription factor). **d**, Overlap between transcription factors that conferred *in vivo* engraftment from hiPSC- and hESC-derived cells injected into mice and *in vitro* multi-lineage CFU potential. Transcription factors detected from genomic DNA PCR from *in vitro* colony screening and *in vivo* engraftment screening are shown. For *in vivo* screening, multiple cell lines (iPSC and ESC) were used. Factors detected by *in vivo* screening were overlapped with those detected by *in vitro* screening (*RUNX1* and *LCOR*). Consistently, *MYB* and *RORA* were detected by *in vitro* screening as previously reported^7^. Overall, *RUNX1*, *LCOR*, *SPI1*, *ERG*, *HOXA5*, *HOXA9*, and *HOXA10* (defined seven transcription factors) were identified in individual experiments with different PSC lines *in vivo*. **e**, Factor-minus-one approach to define essential transcription factors for engraftment. Haemogenic endothelium was infected with combination of seven transcription factors minus one each, as indicated, then transplanted into NSG mice. At the 8 week time point, engraftment of human CD45^+^ cells in bone marrow was determined by FACS. Each panel indicates a representative result of GFP vector: all seven transcription factors, and seven minus *RUNX1*, *ERG*, *SPI1*, *LCOR*, *HOXA5*, *HOXA9*, or *HOXA10* as indicated in the panel. Reduction of chimaerism was seen when *RUNX1*, *ERG*, *LCOR*, *HOXA5*, or *HOXA9* were removed. In contrast, omitting *SPI1* or *HOXA10* had a negligible effect on engraftment.

**Figure 1:**
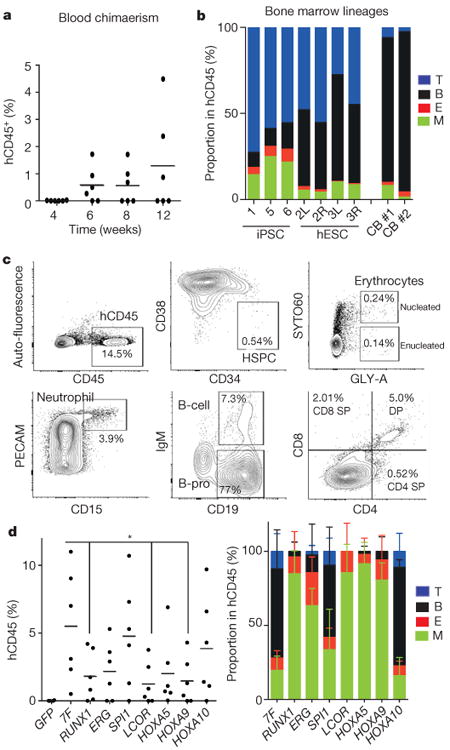
*In vivo* screening identifies transcription factors that enable engraftment from PSCs **a**, Percentage of human CD45^+^ cells detected in peripheral blood of injected mice at indicated number of weeks. **b**, Multi-lineage contribution of human cells in bone marrow of engrafted mice. Bone marrow of NSG mice engrafted with haemogenic endothelium cells infected with the transcription factor library was analysed at 12 weeks for myeloid cells (M; CD33^+^), erythroid cells (E; GLY-A^+^), B cells (CD19^+^), and T cells (CD3^+^) within the human CD45^+^ population. Recipients 1, 5, and 6 were engrafted from hiPSCs; recipient 2 left (L) femur and right (R) femur, recipient 3 left (L) femur and right (R) femur were engrafted from hESCs; recipients CB 1 and CB 2 were engrafted with cord blood HSPCs. **c**, Bone marrow of primary NSG mouse engrafted with HE-7 transcription factor was analysed at 12 weeks for human CD45^+^ HSPCs (CD34^+^CD38^−^), nucleated erythroid cells (GLY-A^+^SYTO60^+^), enucleated erythroid cells (GLY-A^+^SYTO60^−^), neutrophils (PECAM^+^CD15^+^), B cells (IgM^+^CD19^+^), and B progenitor cells (IgM^−^CD19^+^). The thymus was analysed for T cells (CD3^+^/CD4, CD8) (bottom right). **d**, *In vivo* factor-minus-one analysis of defined seven transcription factors to identify necessary and redundant factors. Bone marrow of engrafted NSG was analysed at 8 weeks for human CD45^+^ population. The absence of *RUNX1* (0.33-fold, *P* = 0.037), *ERG* (0.40-fold, *P* = 0.056), *LCOR* (0.23-fold, *P* = 0.020), *HOXA5* (0.37-fold, *P* = 0.056), or *HOXA9* (0.26-fold, *P* = 0.026) reduced chimaerism. Lentiviral vector with green fluorescent protein (GFP) was used as negative control. *N* = 2 mice analysed in two independent experiments with three mice each (two mice each for GFP). *P < 0.05. Average lineage distribution from each group is shown (right). Data shown as mean ± s.d.

We hypothesized that introduction of HSC-specific transcription factors might endow hPSC-derived haemogenic endothelium with the potential to engraft multi-lineage haematopoiesis *in vivo*. Moreover, we reasoned that transcription factors likely to specify haematopoietic stem and progenitor cell (HSPC) fate would be evolutionary conserved. Thus, we interrogated three independent data sets (one mouse^16^ and two human^17,18^) to select 12 transcription factors enriched in fetal-liver HSCs relative to haemogenic endothelium (Extended Data Fig. 2a–c), and selected other candidates from previous reports that used transcription factors to convert endothelial cells^3^, hPSC-derived myeloid cells^7^, or committed lymphoid cells^2^ to haematopoietic progenitor cells. All together, we assembled a library of 26 transcription factors, which were cloned independently into a doxycycline-inducible lentiviral vector. We infected haemogenic endothelium at day 3 of EHT culture at > 50% efficiency (Extended Data Fig. 2d, e). After 24 h we injected the transduced cells intrafemorally into sub-lethally irradiated immune-deficient NOD/LtSz-scidIL2Rγnull (NSG) mice. Mice received doxycycline in their drinking water and diet for 2 weeks to induce transgene expression, after which doxycycline was withdrawn and haematopoietic chimaerism was assessed over time (Extended Data Fig. 2f). We observed increasing chimaerism of human CD45^+^ cells in peripheral blood of injected mice for up to 12 weeks (Fig. 1a). Examination of bone marrow and thymus demonstrated the presence of human erythroid cells (GLY-A^+^), myeloid cells (CD33^+^), B cells (CD19^+^), and T cells (CD3^+^) (Fig. 1b and Extended Data Fig. 2g). From a total number of 40 mice injected in 5 independent experiments with library-infected cells (Extended Data Fig. 3a), 6 recipients engrafted with human induced pluripotent stem cell (hiPSC)-derived haemogenic endothelium and 5 recipients engrafted with human embryonic stem cell (hESC)-derived haemogenic endothelium. Twelve weeks after the injection of the cells, three of the six mice engrafted from iPSCs and two of five mice engrafted from hESCs showed multi-lineage reconstitution of T cells and B cells, as well as myeloid and erythroid cells (Fig. 1b). Human CD34^+^ cord blood HSPCs engrafted in NSG mice as controls showed a predominance of B cells (86 ± 11% from the engrafted human cells) over T cells, as previously described^19^ (Fig. 1b). Notably, after unilateral intrafemoral injection of transduced hESC-derived haemogenic endothelium, we observed comparable human haematopoietic engraftment in both femurs, indicating repopu lation of the contralateral femur through migration and homing of HSPCs (Fig. 1b). Furthermore, we identified a positive correlation between cultures undergoing robust EHT (indicated by RUNX1c-enhancer reporter^20^) and their subsequent ability to engraft multi-lineage haematopoiesis (Extended Data Fig. 3b, c). To confirm the hPSC origin of our engrafted cells, we demonstrated by single nucleotide polymorphism (SNP) array genotyping that human CD45^+^ cells collected from peripheral blood were identical to the input hPSCs (Extended Data Fig. 4a, b). Together, these results demonstrate that infection with a 26-transcription-factor library promotes multi-lineage haematopoietic engraftment from hPSC-derived haemogenic endothelium.

We then determined which of the 26 transcription factors could be detected in the engrafted cells by PCR amplification in sorted populations of human CD33^+^ myeloid cells, CD19^+^ B cells, and CD3^+^ T cells. Seven transcription factors (*ERG*, *HOXA5*, *HOXA9*, *HOXA10*, *LCOR*, *RUNX1*, *SPI1*) were consistently detected in myeloid, B, and T cells of five engrafted recipients, suggesting that these factors conferred multi-lineage haematopoietic reconstitution potential (Extended Data Fig. 4c). These seven transcription factors were recovered both in iPSC and in hESC-derived cells. Additionally, *ZKSCAN1*, *SSBP2*, *MAFF*, *DACH1*, and *SOX4* were detected in some animals, perhaps reflecting their potential to enhance engraftment under some experimental conditions. Distinct factors were recovered when screening for *in vitro* colony-forming potential (Extended Data Fig. 4d).

We next determined whether the seven common transcription factors were necessary and sufficient to support multi-lineage engraftment of haemogenic endothelium *in vivo*. We transduced haemogenic endothelium with these seven transcription factors, injected cells intrafemorally into sub-lethally irradiated NSG mice, and treated the mice with doxycycline for 2 weeks. We observed multi-lineage engraftment in bone marrow and thymus at 12 weeks (Fig. 1c). We sought to determine the minimal combination of transcription factors required for multi-lineage engraftment by a factor-minus-one approach. No exclusion of a single factor abolished engraftment, although omitting *RUNX1*, *ERG*, *LCOR*, *HOXA5*, or *HOXA9* compromised multi-lineage reconstitution and reduced total chimaerism in bone marrow at 8 weeks (Fig. 1d and Extended Data Fig. 4e). These data suggest that, at a minimum, *RUNX1*, *ERG*, *LCOR*, *HOXA5*, and *HOXA9* facilitate engraftment and multi-lineage differentiation.

## Transcription factors confer multi–lineage engraftment

**Extended Data Figure 5:**
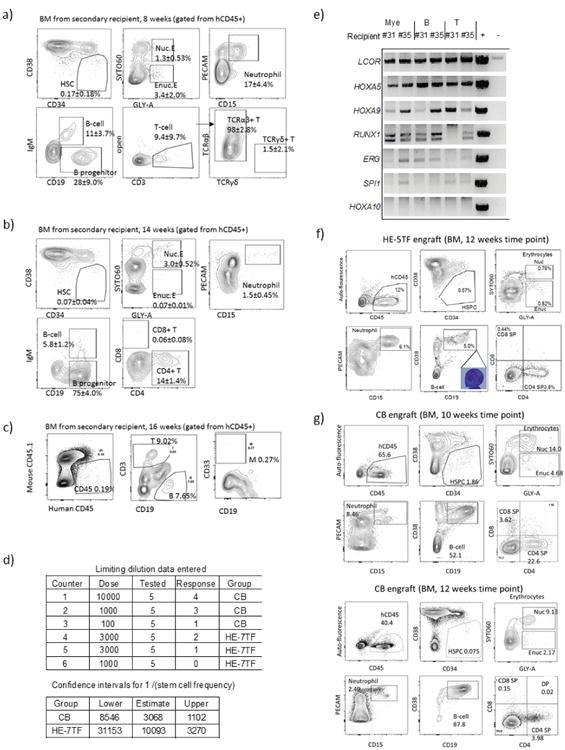
FACS analysis after secondary transplantation of HE-7TF cells Human CD34^+^ cells were obtained by magnetic cell isolation from bone marrow of primary recipients of HE-7TF cells, then 3,000 cells (a, b) or 1,000 cells (c) were intrafemorally transplanted into secondary recipients. Multi-lineage engraftment in bone marrow from a representative recipient mouse is shown; **a**, 8 weeks; **b**, 14 weeks; **c**, 16 weeks. Specific mice analysed are indicated in Fig. 2b. **d**, Limiting dilution assay of HE-7TF cells after secondary transplantation. CD34^+^ cells were isolated from bone marrow of primary recipients, and either 1,000 or 3,000 cells were transplanted into secondary recipients. Multi-lineage engrafted recipients were counted as response. Confidence interval of 1/(stem-cell frequency) was calculated by ELDA (http://bioinf.wehi.edu.au/software/elda/) according to Poisson distribution. A limiting dilution assay of cord blood was used as reference. **e**, Transgene detection in engrafted cells of secondary recipients of HE-7TF cells. Recipient numbers are from Fig. 2b (numbers 31 and 35). **f**, Bone marrow chimaerism of primary mouse engrafted with 5-TF^Poly^ at 12 weeks. Human CD45^+^ bone marrow of engrafted NSG was analysed for HSPCs (CD34^+^CD38^−^), nucleated erythroid (GLY-A^+^SYTO60^+^), enucleated erythroid (GLY-A^+^SYTO60^−^), neutrophils (PECAM^+^CD15^+^), B cells (IgM^+^CD19^+^), B progenitor cells (IgM^−^CD19^+^), B lymphocytes (IgM^−^CD19^+^CD38^++^), and T cells (CD3^+^/CD4, CD8). **g**, Representative FACS plots of bone marrow engrafted with human cord blood HSCs are shown at 10 and 12 weeks.

**Figure 2:**
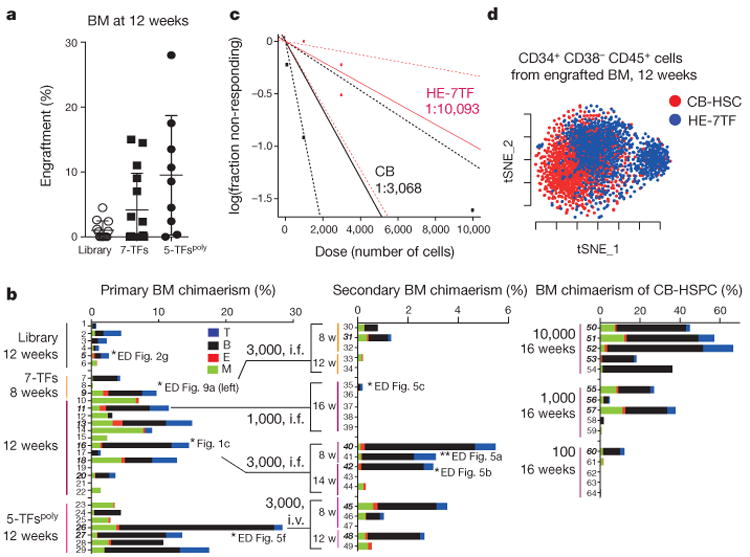
Defined transcription factors confer multi-lineage engraftment **a**, Engraftment of human CD45^+^ cells in bone marrow (BM) of recipient mice detected by flow cytometry at 12 weeks for haemogenic endothelium cells infected with transcription factor library (*n* = 12 mice analysed in two independent experiments with six mice each), seven transcription factors (*n* = 15 mice analysed in five independent experiments with three mice each), and 5-TF^Poly^ (*n* = 9 mice analysed in three independent experiments with three mice each). **b**, Bone marrow lineage distribution of myeloid cells (CD33^+^), erythroid cells (GLY-A^+^), B cells (CD19^+^), and T cells (CD3^+^) is shown as a bar graph for individual mice (numbers 1–29, primary recipients; numbers 30–49, secondary recipients). Each number indicates independent mice at time of bone marrow analysis after being euthanized (w, weeks). Multi-lineage capacity was assessed from bone marrow samples. Right: CD34^+^ cord-blood-engrafted recipients (numbers 50–64) injected with indicated numbers of cells. Secondary recipient numbers 30–34 were transplanted with cells from mouse number 9; numbers 35–39 were transplanted with cells from number 11; numbers 40–44 received cells from number 16; numbers 45–49 received cells from number 26). The dose and route of injected cells is shown (i.f., intrafemoral; i.v., intravenous). Mice numbers set in bold sloping type indicate multi-lineage engraftment with myeloid cells, erythroid cells, B cells, and T cells. Data are shown for a subset of mice. Compiled experience to date is reported in Extended Data Fig. 3. **c**, Limiting dilution assay of HE-7TF cells during secondary transplantation. Three thousand or 1,000 CD34^+^ cells isolated from bone marrow of primary recipients were transplanted to secondary recipients. Multi-lineage engraftment was counted as response. Confidence intervals of 1/(stem cell frequency) were calculated by ELDA (http://bioinf.wehi.edu.au/software/elda/) according to Poisson distribution. Limiting dilution assay of cord blood was used as reference. **d**, *t*-Distributed stochastic neighbour embedding (t-SNE) plot of inDrops single-cell RNA-seq data for CD34^+^CD38-CD45^+^ HSPC populations recovered from bone marrow of mice engrafted with HE-7TF cell- or cord blood HSC-engrafted at 12 weeks. Analysis is anchored on 62 canonical haematopoietic genes (see Methods). Data shown as mean ± s.d.

We monitored mice engrafted with haemogenic endothelium transduced with the defined 7 transcription factors (HE-7TF cells) and documented multi-lineage engraftment with erythroid cells (GLY-A^+^), myeloid cells (CD33^+^), B cells (CD19^+^), and T cells (CD3^+^) in 5 of 13 recipients at 12 weeks. The remaining eight recipients were engrafted with B cells and T cells and either erythroid or myeloid cells (Fig. 2a, b). We next validated the self-renewal capacity of haemogenic endothelium-derived cells by secondary transplantation. We transplanted marrow from three primary mice (9, 11, and 16; Fig. 2b) that showed multi-lineage engraftment at 8 and 12 weeks into secondary animals. Multiple secondary recipients engrafted with multi-lineage haematopoiesis at 8, 14, and 16 weeks (Fig. 2b and Extended Data Fig. 5a–c). To quantify the frequency of these secondary repopulation units, we transplanted secondary recipient mice with 1,000 and 3,000 CD34^+^ cells isolated from the marrow of primary engrafted recipients, and observed multi-lineage engraftment in approximately one-third of animals. Among 10 secondary mice injected with 3,000 CD34^+^ cells from primary recipients of HE-7TF, a total of 3 recipients showed multi-lineage reconstitution in independent experiments, whereas 1,000 CD34^+^ cells from HE-7TF engrafted 0 out of 5 mice (Fig. 2b, c). Using ELDA software, the calculated stem-cell frequency for our HE-7TF cells was 1 in 10,093 cells (Fig. 2c and Extended Data Fig. 5d).

To determine which of the seven transcription factors were consistently recovered from secondary engrafted mice, we performed PCR amplification of myeloid, B and T cells from two mice, and detected *LCOR*, *HOXA5*, *HOXA9*, and *RUNX1* in every lineage. *ERG* was noted in only myeloid and B cells, while *SPI1* and *HOXA10* seemed dispensable (Extended Data Fig. 5e). We then investigated whether the minimal five transcription factors were sufficient to confer multi-lineage engraftment. We engineered two polycistronic lentiviral vectors to transduce iPS-derived haemogenic endothelium with *LCOR-P2A-HOXA9-T2A-HOXA5* and *RUNX1-P2A-ERG*, and detected multi-lineage erythroid cells, neutrophils, B cells, and T cells in multiple engrafted recipients at 12 weeks (5-TFs^poly^; Fig. 2b, left, and Extended Data Fig. 5f, g). Bone marrow transplanted from these mice into irradiated secondary recipients reconstituted multi-lineage haematopoiesis in two of five recipients at 8–12 weeks (Fig. 2b, middle). Our combined experience showed that when haemogenic endothelium was transduced with the entire 26-factor library, 11 out of 40 mice showed engraftment, 5 of which were multi-lineage (3 from iPSC and 2 from hESC). Transduction with 7 transcription factors yielded engraftment in 33 out of 76 mice, with 9 multi-lineage, whereas transduction with 5 transcription factors yielded engraftment in 15 out of 30 mice, with 5 multi-lineage. Engraftment and multi-lineage contribution were assessed from bone marrow at 4–16 weeks (Extended Data Fig. 3).

**Extended Data Figure 6:**
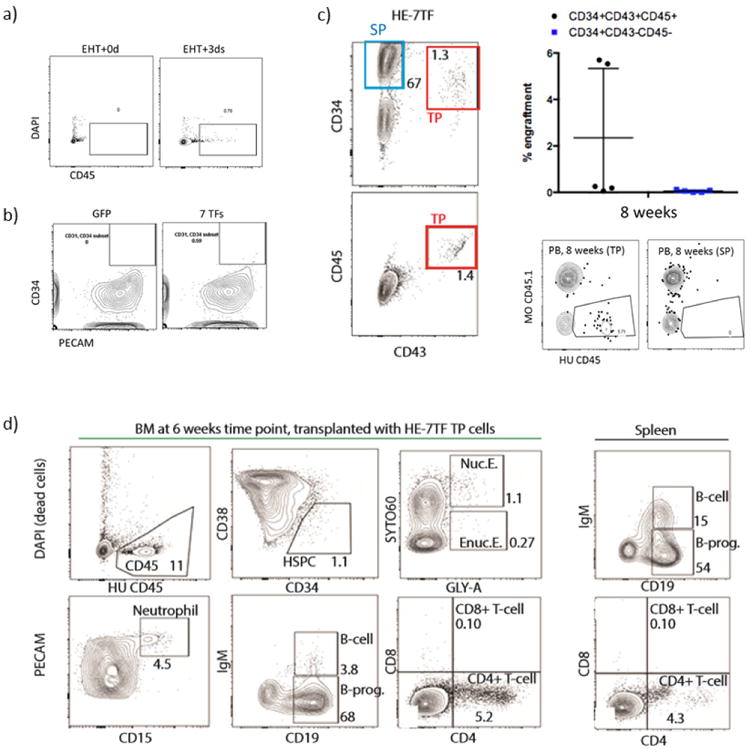
Identification of the source of engraftable cells within the HE-7TF population **a**, Conversion of haemogenic endothelium (HE) into haematopoietic stem/progenitor cells by seven transcription factors requires EHT. Haemogenic endothelium or haemogenic endothelium grown in EHT medium for 3 days was transduced with seven transcription factors and transplanted into mice followed by bone marrow analysis at 4 weeks. Cells grown after 3 days of EHT showed CD45^+^ cells while those that were not grown under EHT conditions did not show CD45^+^ cells. **b**, Human umbilical vein endothelial cells were transduced with seven transcription factors or GFP lentiviral vectors, then cultured in EHT medium with Dox for a week. Flow cytometry analysis of PECAM (EC marker) and CD34 (haematopoietic marker) is shown. Human umbilical vein endothelial cells transduced with seven transcription factors fails to produce robust CD34^+^ cell population. **c**, Twenty-five thousand CD34^+^CD43^+^CD45^+^ (triple positive, TP) or CD34^+^CD43^−^CD45^−^ (single positive, SP) cells were FACS-isolated and transplanted (*N* = 5 mice per group). Engraftment of human CD45^+^ cells was assessed in peripheral blood (PB) at 8 weeks. FACS plots of human CD45 and mouse CD45.1 of TP (left) and single positive (right) transplanted mice at the 8 week time point are shown. **d**, Multi-lineage engraftment of bone marrow and spleen from primary recipient mouse at 6 weeks is shown. CD34^+^CD43^+^CD45^+^ cells were intravenously injected.

To investigate the source of our long-term engraftable cells, we performed the following three sets of experiments. First, we transduced haemogenic endothelium cells on day 0 or day 3 of EHT followed by transplantation into mice, and observed that day 3 EHT cells transduced with seven transcription factors resulted in engraftment, whereas day 0 EHT cells did not (Extended Data Fig. 6a). This emphasizes the role of EHT culture in generating engraftable cells, and suggests that the seven transcription factors promote engraftment not in haemogenic endothelium cells but in the context of cells undergoing EHT. Second, to show the specificity of the seven transcription factors for haemogenic endothelium cells undergoing EHT, we transduced human umbilical vein endothelial cells, but failed to observe the appearance of a CD34^+^ haematopoietic population (Extended Data Fig. 6b). Finally, we transduced our seven transcription factors on day 1 of EHT, when there were no detectable haematopoietic cells^21^ and cultured them for 6 days, after which we sorted CD34^+^CD43^+^CD45^+^ triple-positive cells and CD34^+^CD43^−^CD45^−^ single-positive cells. We transplanted these two cell populations into non-irradiated *c-Kit*-deficient immune-deficient recipients^22^, and monitored engraftment capacity after 8 weeks of transplantation. Two out of five mice transplanted with triple-positive cells showed multi-lineage engraftment, whereas five mice transplanted with single-positive cells did not (Extended Data Fig. 6c, d), suggesting that haemogenic endothelium cells undergoing EHT are the source of long-term engraftment in our system. In summary, these three experiments suggest that haematopoietic cells that emerge from definitive haemogenic endothelium during the *in vitro* process of EHT, when transduced by a core set of transcription factors, obtain the capacity for multi-lineage haematopoietic reconstitution in recipient mice.

**Extended Data Figure 7:**
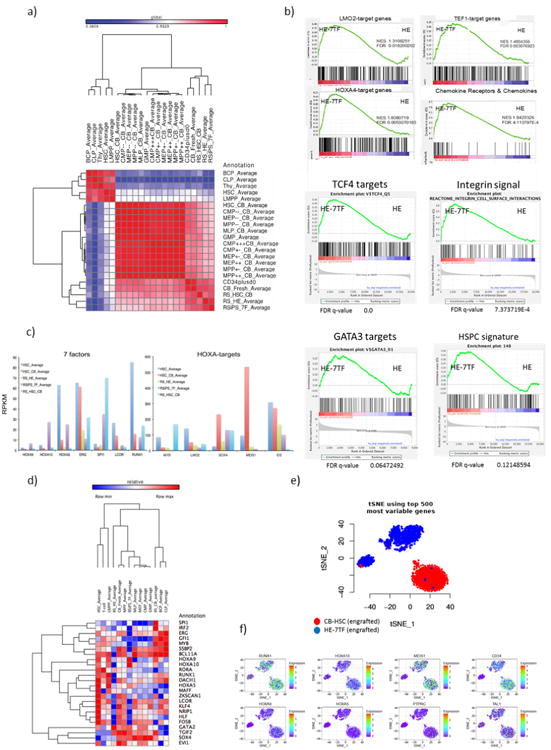
Molecular features of HE-7TF cells **a**, Correlation matrix of RNA-seq data from HSPC populations (CD34^+^CD38^−^CD45^+^) from HE-7TF cells or cord blood HSC engrafted for 12 weeks, iPS-haemogenic endothelium, and publicly available gene expression data (PubMed identifiers 26541609 and 26502406). RNA-seq samples from this study were RSips_7F_Average, RS_HE_Average, and RS_HSC_CB. Samples from ref. 23 were BCP_Average, CLP_Average, Thy_Average, HSC_Average, LMPP_Average, and HSC_CB_Average. The remaining samples are from ref. 24. **b**, Gene set enrichment analysis signature of HE-7TF cells compared with haemogenic endothelium cells. HE-7TF cells show gene expression signatures that positively correlate with LMO2 targets, TEF1 targets, HOXA4 targets, chemokine receptors and chemokines, TCF4 targets, integrin signal, GATA3 targets, and HSPCs. *P* < 0.05 and FDR *q* value < 0.25 were considered significant conditions. All gene set enrichment analysis plots satisfied these conditions, except the HSPC signatures, which had a *q* value of 0.121 and a *P* value of 0.06, suggesting that transcriptional differences remain between HE-7TF and bona fide HSCs/HSPCs. HSPC signature taken from ref. 56. **c**, RPKM values of seven transcription factors and HOXA target genes in indicated cell types are shown. HOXA target genes from ref. 28. PubMed identifier 27183470. **d**, Heatmap depiction of relative expression levels of the 26 transcription factors in the library in the following samples: HE, HE-7TF cells (engrafted), cord blood HSCs (engrafted), and fresh HSCs and progenitors. Notably, HE-7TF cells show high expression of HOXA family genes, *GATA2*, *TGIF2*, *SOX4*, and *EVI1*. **e**, The t-SNE of in-droplet single-cell RNA-seq of HE-7TF cells and cord blood HSCs (engrafted CD34^+^CD38^−^CD45^+^ cells from bone marrow at 12 weeks). The t-SNE from the top 500 most variable genes is presented in the top panel. **f**, Expression value of 8 haematopoietic genes in the same plot. Notably, the middle population (a subpopulation of HE-7TF cells) shared similar expression values, and degrees of heterogeneity of *RUNX1*, *MEIS1*, *CD34*, *TAL1* with cord blood HSCs.

To examine the extent to which the HE-7TF cells recapitulated HSC or progenitor-intrinsic gene expression programs, we compared RNA sequencing (RNA-seq) data from CD34^+^CD38^−^CD45^+^ HSPC populations harvested from bone marrow after 12 weeks of engraftment to publicly available data sets for HSCs and progenitors^23,24^. Global transcriptomic analysis showed that HE-7TF cells strongly correlated with haemogenic endothelium, engrafted cord blood HSCs, and fresh cord blood CD34^+^ cells (Pearson correlation > 0.7; Extended Data Fig. 7a). HE-7TF cells clustered closest to the haemogenic endothelium cells, thereby implying incomplete conversion of HE-7TF cells to an HSC-like state (Extended Data Fig. 7a). Furthermore, gene set enrichment analysis indicated a positive correlation with signatures associated with transcription factors required for specification and/or maintenance of HSCs (Extended Data Fig. 7b). Gene expression signatures correlated with activation of chemokine receptor signalling, and integrin signalling, pathways known to influence HSC homing and engraftment^25–27^. In summary, HE-7TF cells have activated specific transcription factor modules and pathways that reflect their capacity for multi-lineage haematopoietic engraftment and reconstitution in transplant recipients.

Furthermore, we observed that the expression levels of *HOXA5, HOXA9, HOXA10*, and *SPI1* transgenes in HE-7TF cells were comparable to those in HSCs (Extended Data Fig. 7c), whereas the expression of *LCOR, RUNX1*, and *ERG* was suppressed (Extended Data Fig. 7c), indicating that the converted HSPC-like cells partly reprogram only certain loci to establish expression patterns characteristic of native HSCs. Recently, it was shown that the overexpression of *HOXA* genes in iPS-HPCs failed to adequately activate *HOX* target genes^28^, but in the case of HE-7TF cells, multiple *HOXA* target genes were successfully activated including *SOX4* and *ID2* (Extended Data Fig. 7c, right), highlighting the likely context-dependent expression of co-factors that are present in HE-7TF cells. Activation of *HOX* target genes also confirms the comparable expression level of *HOX* genes in the engrafted HE-7TF cells compared with other HSC/HSPCs, as well as upregulation of other HSC-specific transcription factors (*GATA2, TGIF2, SOX4, EVI1*) (Extended Data Fig. 7d). To understand the heterogeneity of HE-7TF cells compared with cord blood HSCs, we performed Indrops single-cell RNA-seq on HE-7TF and cord blood HSCs^29^. Comparing the 500 most variable genes, we observed distinct clustering of HE-7TF and cord blood HSCs (Extended Data Fig. 7e, f); however, when we restricted our analysis to 62 canonical haematopoietic genes, we noted substantial overlap between cord blood HSCs and HE-7TF cells (Fig. 2d). Overall, these data demonstrate that HE-7TF cells recapitulate gene expression signatures consistent with their potential for multi-lineage haematopoietic engraftment, yet remain molecularly distinct from cord blood HSCs.

## Characterization of differentiated cells

**Extended Data Figure 8:**
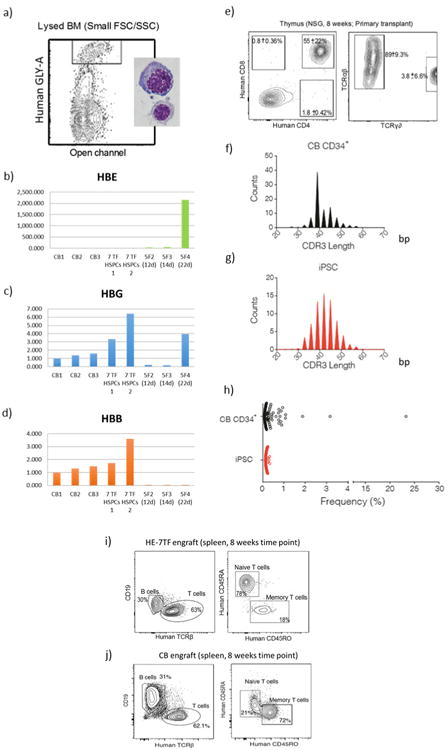
Characterizations of differentiated cells: analysis of definitive erythropoiesis by relative quantification of globin transcripts **a**, Human GLY-A^+^ cells were isolated from lysed bone marrow (to exclude enucleated cells) and analysed by qRT–PCR to quantify (**b**) HBE, (**c**) HBG, and (**d**) HBB genes. CB, GLY-A^+^ erythroid cells from cord-blood-engrafted in NSG bone marrow; 7 TF HSPCs, GLY-A^+^ erythroid cells from seven transcription factor HSPC-engrafted in NSG bone marrow; 5F, GLY-A^+^ erythroid cells from hPSCs transduced with ERG, RORA, HOXA9, SOX4, and MYB^7^. Analysis of T-cell receptor diversity in engrafted T cells. **e**, Flow cytometric phenotyping of T cells from engrafted HE-7TF cells. Thymus was collected at 8 weeks and analysed for T-cell markers (CD4, CD8, CD3, TCRαβ, and TCRγδ). TCR phenotyping of the CD3^+^ population is shown on the right. One out of three recipients showed the presence of TCRγδ. Three thymic engrafted mice from independent experiments each. **f–h**, TCR rearrangement of thymocytes from cord blood CD34^+^ and HE-7TF engrafted in NSG. CD3^+^ T cells were isolated from NSG mice engrafted with (**f**) cord blood HSCs or (**g**) HE-7TF cells. Purified DNA was subjected to next-generation sequencing of the CDR3 using immunoSEQ (Adaptive Biotechnology) and analysed with the immunoSEQ Analyzer software (Adaptive Biotechnology). A high degree of combinatorial diversity in the V-gene segment usage was observed in CDR3 length, following a standard Gaussian distribution. h, Frequency of clonotype of T cells. **i**, Flow cytometric phenotyping of spleens from engrafted HE-7TF cells versus cord blood (**j**). Spleens were collected at 8 weeks and human CD45^+^ cells were analysed for T-cell markers (TCRβ, CD4, CD45RO, and CD45RA), and B-cell marker (CD19).

**Extended Data Figure 9:**
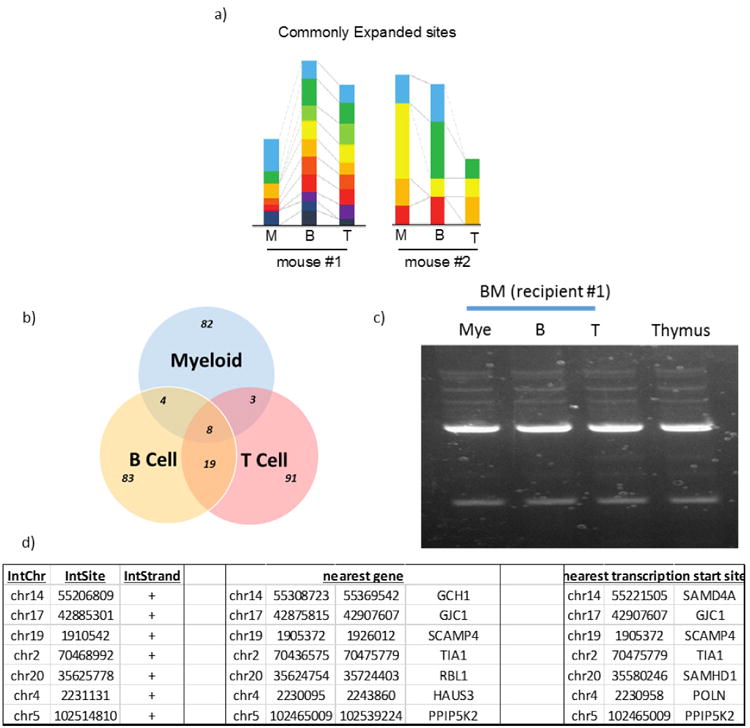
Integration sequencing analysis of engrafted myeloid cells, B cells, and T cells from two individual animals **a**, Left: from 8 weeks, indicated in Fig. 2b; right: from 5 weeks. Clonally expanded populations are shown for each lineage, with common clones among three lineages represented by colour. The smallest proportional coloured segments for each animal represent the unit value of two clonal sequences. **b**, Overlap of commonly expanded integration sites from an animal shown on the left in a. Genomic DNA-sequencing of CD33^+^ myeloid cells, CD19^+^ B cells, and CD3^+^ T cells from bone marrow detected common expanded integration sites. **c**, Agarose gel electrophoresis of adaptor-ligation of engrafted cells. Cells were obtained independently from those used in a at the 1 week time point. **d**, Integrated loci mapped in genome. Nearby genes of common integrated sites are described. These data are from an animal indicated in a at the 8 week time point.

**Figure 3:**
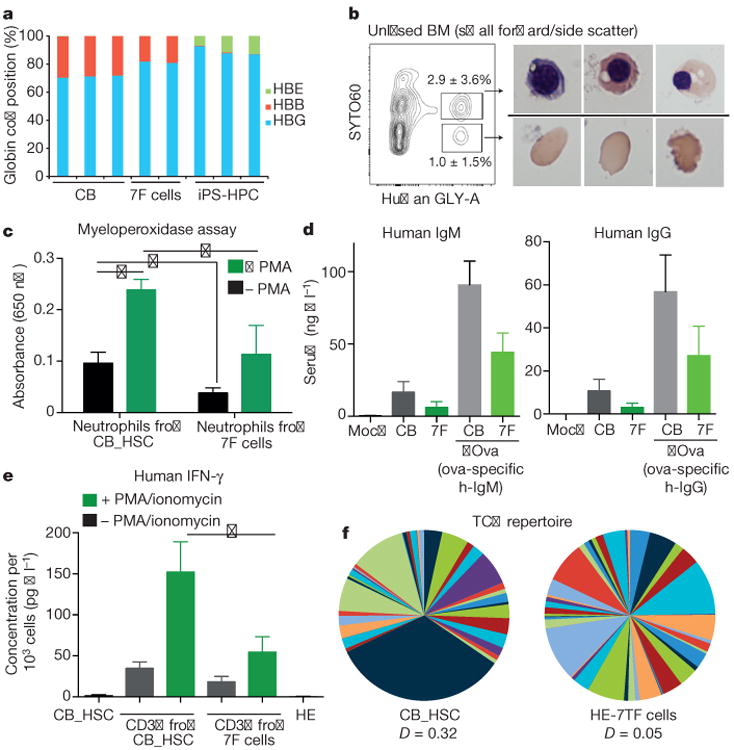
Characterization of differentiated haematopoietic cells in engrafted mice **a**, Adult β -globin expression in engrafted erythroid cells. Human GLY-A^+^ cells were isolated from bone marrow of NSG mice engrafted with HE-7TF cells at 8 weeks, and analysed by quantitative PCR with reverse transcription (qRT-PCR) to quantify embryonic (HBE), fetal (HBG), and adult β -globin (HBB) transcripts. CB, GLY-A^+^ erythroid cells from cord-blood-engrafted in NSG bone marrow; 7F cells, GLY-A^+^ erythroid cells from HE-7TF cell-engrafted in NSG bone marrow; iPS-HPC, GLY-A^+^ erythroid cells from hPSCs transduced with ERG, RORA, HOXA9, SOX4, and MYB^7^. **b**, Enucleation of engrafted erythroid cells. Bone marrow of NSG mice engrafted with HE-7TF cells at 8 weeks was analysed for human GLY-A and SYTO60. Representative cytospin images of red blood cells from GLY-A^+^ populations separated by SYTO60 nuclear staining are shown. *N* = 9 measurements from three independent experiments performed each with three technical replicates. **c**, Neutrophils. Human CD45^+^ cells from bone marrow of NSG mice engrafted with HE-7TF cells at 8 weeks, analysed for human PECAM and CD15. Myeloperoxidase activity of isolated CD45^+^ PECAM^+^ CD15^+^ neutrophils was measured with or without PMA stimulation. Neutrophils from NSG engrafted with cord blood HSCs were used as reference. The basal level of MPO of haemogenic endothelium was 0.40-fold less than cord blood (*P* = 0.036). PMA stimulation increased MPO production 2.5-fold (*P* = 0.010) (cord blood) and 3.0-fold (*P* = 0.10) (seven transcription factor). Stimulated MPO production of HE-7TF was 0.47-fold (0.049) versus cord blood. *P < 0.05. *N* = 6 measurements from two independent experiments performed each with three technical replicates. **d**, Human immunoglobulin. Serum was isolated from NSG mice engrafted with cord blood HSCs or HE-7TF cells at 8 weeks and 14 weeks. Production of IgM (8 weeks) and IgG (14 weeks) was measured by enzyme-linked immunosorbent assay (ELISA). Serum from mock transplant and NSG engrafted with cord blood HSCs was used as reference. Human ovalbumin-specific IgM and IgG after immunization with ovalbumin (ova) in serum of mice engrafted with HE-7TF cells, as indicated (right two bars of each panel). *P < 0.05. *N* = 6 measurements from two independent experiments (three for cord blood versus HE) performed with three technical replicates. **e**, Production of IFN-γ from human CD3^+^ cells isolated from bone marrow of NSG mice engrafted with cord blood HSCs and HE-7TF cells at 8 weeks, and cultured with or without PMA/ionomycin stimulation for 6 h. CD3^+^ T cells from NSG engrafted with cord blood HSCs were used as reference. The basal level of IFN-γ of haemogenic endothelium was 0.53-fold (*P* = 0.073) versus cord blood. PMA stimulation increased IFN-γ production 4.4-fold (*P* = 0.17) (cord blood) and 3.0-fold (*P* = 0.16) (haemogenic endothelium). Stimulated IFN-γ production of haemogenic endothelium was 0.36-fold (0.039) versus cord blood. IFN-γ production from cord blood HSCs and haemogenic endothelium themselves are shown as reference. *P <0.05. *N* = 6 measurements from two independent experiments performed with three technical replicates. **f**, TCR repertoire of engrafted T cells. Human CD3^+^ thymocytes of NSG mice engrafted with HE-7TF cells at 8 weeks were analysed by immunoSEQ to detect TCR rearrangement. CD3^+^ thymocytes from cord blood HSC-engrafted NSG were used as a reference.

We examined erythroid, myeloid, and lymphoid cells recovered from engrafted mice, and compared their phenotypic and functional properties to those of cord blood HSC-derived cells. Definitive erythropoiesis is characterized by globin switching and enucleation^30^. Most erythroid cells generated from hPSCs express embryonic and fetal globins and retain nuclei^7^, whereas human erythroid cells recovered from our engrafted mice lacked expression of embryonic *HBE*, and expressed fetal *HBG* and adult *HBB* at levels comparable to colony-forming unit-erythroid (CFU-E) cells from human cord blood (Fig. 3a and Extended Data Fig. 8a–d). Human GLY-A^+^ cells derived from HE-7TF cells underwent enucleation, indicating developmental maturation of the engrafted human erythroid cells into definitive erythrocytes (Fig. 3b). To measure the response of human myeloid cells in NSG recipients to cytokine stimuli by activation of myeloperoxidase (MPO), we isolated human CD45^+^CD15^+^PECAM^+^ neutrophils from engrafted bone marrow, and compared MPO production to engrafted cord-blood-derived cells. Phorbol myristate acetate (PMA) stimulation enhanced the production of MPO three fold relative to unstimulated neutrophils (Fig. 3c). Bona fide human HSCs generate functional T and B cells in NSG mice^19^. In the sera of NSG mice engrafted with HE-7TF cells, we detected human immunoglobulin-M and -G (IgM and IgG), indicating cooperative activity of T and B cells in mediating immunoglobulin class switching and antibody secretion (Fig. 3d). Moreover, inoculation with ovalbumin protein boosted levels of ovalbumin-specific IgM and IgG, indicating a functional immune response to a protein antigen (Fig. 3d). We isolated mature CD3^+^ T cells from bone marrow and observed interferon-γ (IFN-γ) production after re-stimulation using PMA/ionomycin (Fig. 3e). T cells develop from CD4^−^CD8^−^ double-negative cells followed by CD4^+^CD8^+^ double-positive cells that express surface T-cell antigen receptor (TCR)/CD3 complex, which differentiate to either CD4 or CD8 single-positive T cells in the thymus, and then migrate to blood and bone marrow^31^. At 8 weeks, HE-7TF cell-derived thymocytes were predominantly CD4^+^CD8^+^ (55 ± 22%), with few CD4^+^CD8^−^ (1.8 ± 0.42%) and CD4^−^CD8^+^ (0.80 ± 0.36%) cells (Extended Data Fig. 8e). Human CD3^+^ T cells differentiated from cord blood HSCs in NSG mice possess either TCRαβ (>60%) or TCRγδ (<30%)^19^. HE-7TF cell-derived grafts produced most TCRαβ (89 ± 9.3%) and only low levels of TCRγδ (3.8 ± 6.6%) cells (Extended Data Fig. 8e). Development of a diverse population of antigen-specific T cells requires rearrangement of germline-encoded *TCR* genes^32^, largely mediated by recombination of the complementarity determining region 3 (*CDR3*) within variable (V) gene segments of the *TCRA* and *TCRB* genes. To determine clonotype diversity, we profiled the *CDR3* region of *TCRB* on CD3^+^ T cells isolated from reconstituted mice using next-generation sequencing. We observed a high degree of combinatorial diversity in the V-gene segment usage of CD3^+^ T cells isolated from either cord-blood-engrafted NSG or HE-7TF cell-engrafted NSG mice, with the *CDR3* length following a standard Gaussian distribution (Fig. 3f and Extended Data Fig. 8f–h). In addition, splenocytes isolated from engrafted mice harboured human B cells, naive T cells, and memory T cells (Extended Data Fig. 8i versus cord blood engraft in Extended Data Fig. 8j). We then determined whether the myeloid and lymphoid progeny of HE-7TF cells were of clonal origin by comparing sequences of lentiviral integration sites^33^. Genomic DNA from myeloid (CD33^+^), B (CD19^+^), and T cells (CD3^+^) from bone marrow and thymus at 5 and 8 weeks after transplantation was sonicated, followed by adaptor-ligation PCR and deep sequencing (8-week-old mouse used is indicated in Fig. 2b). Among all recovered integration sites, those common to myeloid cells, B cells, and T cells represented up to 10% of the total and were found in each individual recipient (Extended Data Fig. 9a–c), indicating that reconstitution is driven, at least in part, by single clonal HSC-like cells with multi-lineage potential. None of the genes adjacent to integrated proviruses have previously been linked to clonal expansion in previous HIV/AIDS studies (Extended Data Fig. 9d).

## Discussion

**Extended Data Figure 10:**
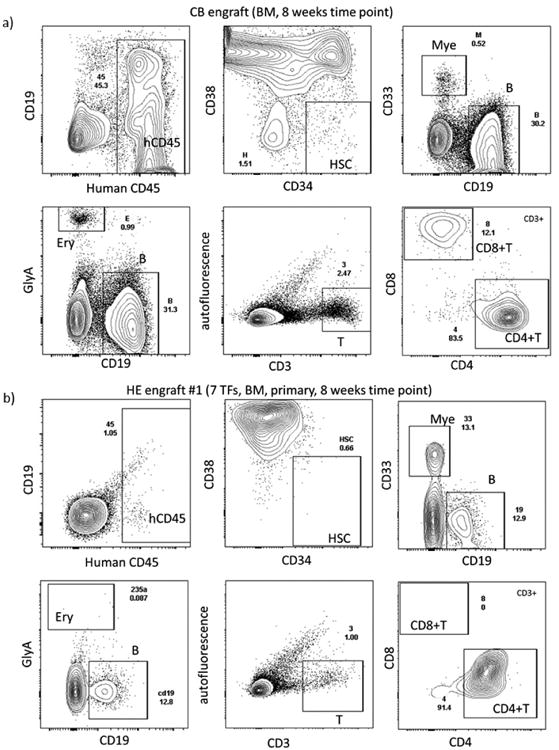
Representative FACS plots **a, b**, FACS plot of cord blood HSCs (**a**) and HE-7TF cell-engrafted NSG mice at the 8 week time point (**b**). Each panel shows human CD45^+^ engraftment, HSPCs (CD34^+^CD38-), myeloid cells (CD33^+^), B cells (CD19^+^), T cells (CD3^+^CD4^+^CD8^+^), and erythrocytes (GLY-A^+^). Spleens were collected at 8 weeks, and human CD45^+^ cells were analysed for T-cell markers (TCRβ, CD4, CD45RO and CD45RA), and B-cell marker (CD19).

The generation of functional HSC-like cells from PSCs has been a long-sought goal in haematology research. By directed differentiation of hPSCs to haemogenic endothelium followed by *in vivo* screening of transcription factors for haematopoietic progenitor specification, we have identified seven transcription factors that together confer HSC-like engraftment, self-renewal, and multi-lineage capacity. Although there is still a molecular and functional gap between our engineered cells and bona fide HSCs in their robustness of engraftment and full recapitulation of terminally differentiated cells (summarized in Extended Data Fig. 10 and Supplementary Table 1), our combined approach of morphogen-driven differentiation and transcription-factor-mediated cell fate conversion produces HSPCs from PSCs.

Combinations of transcription factors have been introduced into differentiated blood cells^2,7^ to endow HSC-like properties in a mouse system^2^, but transplantable human HSCs with multi-lineage capacity and immunological functionality have so far not been derived from hPSCs. Recent advances in the directed differentiation of PSCs to definitive haemogenic endothelium have provided a supportive context for screening of HSC-specifying transcription factors. Each of the identified transcription factors in our study plays a role in HSC development, maintenance of long-term HSCs, or lineage commitment. RUNX1 is essential for haematopoietic commitment of haemogenic endothelium and can convert endothelial cells to haematopoietic progenitor cells^3,34,35^. LCOR, a component of a histone deacetylation complex, is mutated in B-cell lymphoma, suggesting a role in B-lymphopoiesis^36,37^, but this factor has not previously been implicated in HSC function and its role remains to be defined. SPI1 (also known as PU.1) is required for haematopoietic progenitor cell emergence and regulates myeloid specification^38^. HOX family members have been reproducibly implicated in haematopoiesis across species^39,40^. HOXA9 is the key homeotic gene that defines HSC identity^41,42^, interacting with ERG to support HSC renewal during embryogenesis and stress haematopoiesis^43–45^, suggesting a basis for the functional cooperation of HOXA9 and ERG in our system. HOXA5 is a transcriptional target of Notch signalling in T-cell progenitors along with HOXA9 and HOXA10, consistent with a role in T-lymphopoiesis^46^. These factors share binding sites in the genome and cooperate to recruit chromatin modulators (for example, RUNX1 and HOXA families)^45,47^ to induce and maintain HSPCs.

Our factor-minus-one approach to the defined seven transcription factors suggested that they each contribute to a common gene regulatory network with some redundancy, as exclusion of individual factors did not fully abrogate engraftment.

Our study suggests that we are tantalizingly close to realizing the potential of derivation of HSC-like cells from PSCs. Such cells, when derived from patients with genetic blood disorders, offer considerable promise for modelling human blood disease, for humanizing mice for research applications, and for testing the capacity of gene therapy vectors or pharmacological agents to restore haematopoietic function. Our ultimate goal remains the derivation of bona fide transgene-free HSCs for applications in research and therapy.

## Online Content

Methods, along with any additional Extended Data display items and Source Data, are available in the online version of the paper; references unique to these sections appear only in the online paper.

## Methods

A step-by step protocol describing the HSPC conversion of human PSCs can be found at Protocol Exchange^48^.

### hPSC culture, registration, and deposition of hPSC lines

All experiments were performed with H9 hESC (NIHhESC-10-0062), PB34 iPS^49^, MSC-iPS1^50^, 1045-iPSC, and 1157-iPSC established by the hES Core Facility at Boston Children's Hospital. Human ESCs and iPSCs were maintained on hESC-qualified Matrigel (BD) in mTeSR1 media (Stem Cell Technologies) or mouse embryonic fibroblasts (GlobalStem) feeders in DMEM/F12 + 20% KnockOutSerum Replacement (Invitrogen), 1 mM l-glutamine, 1 mM NEAA, 0.1 mM β-mercaptoethanol, and 10 ng ml^−^1 bFGF on 10 cm gelatinized culture dishes. Medium was changed daily and cells were passaged 1:4 onto fresh feeders every 7 days using standard clump passaging with dispase. Morphology of PSCs was checked by microscopy daily. As a quality control, only dishes with more than 70% of typical PSC colonies were processed for embryoid body formation. Cell lines were tested for mycoplasma routinely.

### Embryoid body differentiation

Embryoid body differentiation was performed as previously described^19^. Briefly, hPSC colonies were dissociated with 0.05% trypsin for 5 min at 37 °C, pipetted thoroughly with p1000 to form small aggregates, washed twice with PBS + 2% FBS, and resuspended in StemPro-34 (Invitrogen, 10639-011) supplemented with l-glutamine (2 mM), penicillin/streptomycin (10 ng ml^−1^), ascorbic acid (1 mM), human holo-Transferrin (150 μg ml^−1^, Sigma T0665), mono-thioglycerol (MTG, 0.4 mM) (referred to as ‘supplemented StemPro-34’), BMP4 (10 ng ml^−1^), and Y-27632 (10 μM). Cells were then seeded into non-adherent spheroid formation 10 cm plates (Ezsphere, Asahi Glass; well size diameter 400–500 μm, depth 100–200 μm; number of wells 14,000 per dish) at a density of 5 million per dish. Twenty-four hours later, bFGF (5 ng ml^−1^) and BMP4 (10 ng ml^−1^) were added to the medium. On day 2, the developing embryoid bodies were collected and resuspended in supplemented StemPro-34 with SB431542 (6 μM), CHIR99021 (3 μM), bFGF (5 ng ml^−1^), and BMP4 (10 ng ml^−1^). The formation of embryoid bodies was checked by microscopy on day 4 and the decision was made to continue embryoid body formation on the basis of the size and morphology of aggregations (quality control; > 100 μM, compaction-like tight contact of cells). On day 4, medium was replaced by supplemented StemPro-34 with VEGF (15 ng ml^−1^) and bFGF (10 ng ml^−1^). At day 6, medium was replaced by supplemented StemPro-34 with bFGF (10 ng ml^−1^), VEGF (15 ng ml^−1^), interleukin (IL)-6 (10 ng ml^−1^), IGF-1 (25 ng ml^−1^), IL-11 (5 ng ml^−1^), and SCF (50 ng ml^−1^). Cultures were maintained in a 5% CO_2_/5% O_2_/90% N_2_ environment. All recombinant factors were human and purchased from Peprotech.

### Haemogenic endothelium sorting

To avoid potential damage resulting from hydrodynamic pressure and contamination through fluorescence-activated cell sorting (FACS), for functional assay, isolation of haemogenic endothelium was performed by magnetic cell isolation. Freshly dissociated embryoid body cells (at the day 8 time point) by 0.05% trypsin were filtered through a 70 μm filter and stained with CD34 microbeads (Miltenyi) for 30 min at 4 °C. CD34^+^ cells were isolated with LS columns (Miltenyi). Around 0.3 × 10^5^ to 1.0 × 10^5^ cells were obtained per 10 cm dish of embryoid body formation. A sample from each batch was analysed by FACS to validate its purity of haemogenic endothelium with the panel CD34 PE-Cy7 (8G12; BD), FLK1 AF647 (89106; BD), CD235a/glycophorin (GLY)-A FITC (11E4B-7-6; Coulter), CD43 PE (1G10; BD), and 4′,6-diamidino-2-phe-nylindole (DAPI). For expression profiling by microarray and qRT–PCR, isolation of haemogenic endothelium was performed by FACS. Dissociated embryoid bodies (at the day 8 time point) were resuspended at 1 × 10^6^ to 3 × 10^6^ per 100 μl of staining buffer (PBS + 2% FBS). Cells were stained with a 1:50 dilution of CD34 PE-Cy7 (8G12; BD), FLK1 AF647 (89106; BD), CD235a/glycophorin (GLY)-A FITC (11E4B-7-6; Coulter), CD43 PE (1G10; BD), and DAPI for 30 min at 4 °C in the dark. All FACS sorting was performed on a BD FACS Aria II cell sorter using an 85 μm nozzle to avoid potential damage to haemogenic endothelium.

### Microarray analysis of haemogenic endothelium

All the samples used for microarray analysis were FACS-sorted. Haemogenic endothelium panel: CD34 PE-Cy7 (8G12; BD), FLK1 AF647 (89106; BD), CD235a/glycophorin (GLY)-A FITC (11E4B-7-6; Coulter), and CD43 PE (1G10; BD). Fetal-liver HSCs were purchased from StemCell Technologies and stained with HSC panel: CD38 PE-Cy5 (LS198-4-3; Clontech), CD34 PE-Cy7 (8G12; BD), and CD45 PE (HI30; BD). Between 10,000 and 50,000 cells were sorted for each cell type with two or three biological replicates. An RNAeasy Microkit (Qiagen) was used to collect and prepare total RNA for microarray analysis. The Ovation Picokit (Nugen) was used for preamplification, where required. Gene expression profiling was performed on Affymetrix 430 2.0 gene chips according to standard protocol. Microarray data were analysed according to standard protocol using R/Bioconductor.

### EHT culture

Embryoid bodies were dissociated on day 8 by digestion with 0.05% trypsin for 5 min at 37 °C, pipetted thoroughly with p1000 to generate a single-cell suspension and washed with PBS + 2%FBS. Dissociated embryoid bodies were immediately processed for isolation of haemogenic endothelium. Cells were resuspended in 1 mL PBS^+^2%FBS and incubated with human CD34 MicroBead kit for 1 h (Miltenyl Biotec, 130-046-702). After incubation, cells were washed with PBS^+^2%FBS and isolated by magnetic cell isolation using LS columns (Miltenyl Biotec, 130-042-401). Sorted CD34^+^ cells were resuspended in supplemented StemPro-34 medium, containing Y-27632 (10 μM), TPO (30 ng ml^−1^), IL-3 (10 ng ml^−1^), SCF (50 ng ml^−1^), IL-6 (10 ng ml^−1^), IL-11 (5 ng ml^−1^), IGF-1 (25 ng ml^−1^), VEGF (5 ng ml^−1^), bFGF (5 ng ml^−1^), BMP4 (10 ng ml^−1^), and FLT3 (10 ng ml^−1^) as reported^20^ and seeded at a density of 25 × 10^3^ to 50 × 10^3^ cells per well onto thin-layer Matrigel-coated 24-well plates. All recombinant factors were human and most were purchased from Peprotech.

### Lentivirus production

Plasmids for the transcription factor library were obtained as Gateway plasmids (Harvard Plasmid Service; GeneCopoeia). Open reading frames were cloned into lentiviral vectors using LR Clonase (Invitrogen). Two vectors were used, pSMAL-GFP (constitutive) and pINDUCER-21 (ORF-EG)^51^. pINDUCER21 (ORF-EG) was a gift from S. Elledge and T. Westbrook (Addgene plasmid 46948). Lentiviral particles were produced by transfecting 293T-17 cells (ATCC) with the second-generation packaging plasmids (pMD2.G and psPAX2 from Addgene). Virus were harvested 36 and 60 h after transfection and concentrated by ultracentrifugation at 23,000 r.p.m. for 2 h 15 min at 4 °C. Viruses were reconstituted with 50 μl of EHT culture medium. Constructs were titred by serial dilution on 293T cells using GFP as an indicator. Polycistronic vectors were made as follows: LCOR-P2A-HOXA9-T2A-HOXA5 and RUNX1-P2A-ERG DNA fragments were synthesized and cloned into pENTR-D/TOPO cloning vector by GenScript, then Gateway-recombined with pINDUCER-21 (ORF-EG).

### Lentiviral gene transfer

At day 3 of EHT culture, haemogenic endothelium cells were beginning to produce potentially haematopoietic ‘round’ cells; the occurrence of this phenomenon was used as quality control of haemogenic endothelium induction and transition to haematopoietic cells for each batch of experiments. The infection medium was EHT culture medium supplemented with Polybrene (8 μg ml^−1^, Sigma). Lentiviral infections were performed in a total volume of 250 μl (24-well plate). The multiplicity of infection for the factors was as follows: Library 3.0 for each, ERG 5.0, HOXA5 5.0, HOXA9 5.0, HOXA10 5.0, LCOR 5.0, RUNX1 5.0, SPI1 5.0, LCOR–HOXA9–HOXA5 2.0, and RUNX1-ERG 2.0. Haemogenic endothelium was vulnerable to damage during spinoculation, thus infections were performed static for 12 h, then 250 μl of fresh EHT medium was supplemented to dilute Polybrene. Parallel wells were cultured for an additional 3 days to measure infection efficiency by the percentage of GFP^+^ DAPI cells by FACS, achieving 30–70% of infection efficiency.

### *In vitro* screening via CFU

Followed by lentiviral gene transfer, cells were maintained for 5 days in EHT culture medium supplemented with doxycycline (2 μg ml^−^1, Sigma) to induce transgene expression *in vitro*. Fifty thousand cells were plated into 3 ml complete methylcellulose (H4434; StemCell Technologies). Additional cytokines added were 10 ng ml^−1^ FLT3, 10 ng ml^−1^ IL6, and 50 ng ml^−1^ TPO (R&D Systems). The mixture was distributed into two 60 mm dishes and maintained in a humidified chamber at 37 °C for 14 days. Colonies were scored manually or using a BD Pathway 855 fluorescent imager. At 14 days, granulocyte, erythrocyte, monocyte, megakaryocyte (GEMM) colonies were picked up by P20 pipette. Between 10 and 20 GEMM colonies were picked with 2 or 3 biological replicates. A QIAamp DNA Micro Kit (Qiagen) was used to collect and prepare total genomic DNA for PCR detection of transgenes. Nested PCR reaction was as follows: first round with LNCX forward primer (5′-AGC TCG TTT AGT GAA CCG TCA GAT C-3′) and EGFP N reverse primer (5′-CGT CGC CGT CCA GCT CGA CCA G-3′), 95 °C 5 min, 36 cycles of (95 °C for 30 s, 60 °C for 30 s, 72 °C for 5 min), 72 °C for 5 min, 4 °C hold; second round with forward primer for each gene and HA reverse primer (5′-TCT GGG ACG TCG TAT GGG TA-3′), 95 °C 5 min, 36 cycles of (95 °C for 30 s, 60 °C for 30 s, 72 °C for 30 s), 72 °C for 5 min, 4 °C hold.

### *In vivo* screening via transplantation

Twelve hours after lentiviral gene transfer, cells were recovered by dispase for 5 min at 37 °C, and washed by PBS three times to ensure no carry-over of virus. Cells were resuspended at 0.3 × 10^5^ to 3.0 × 10^5^ cells per 25 μl buffer (PBS + 2% FBS from StemCell Technologies) and kept on ice until injection. Thirty thousand to 3.0 × 10^5^ cells were intrafemorally injected in to NOD/LtSz-scidIL2Rgnull (NSG) mice and treated with doxycycline as described below (see section on ‘Mouse transplantation’). Up to 100 μl peripheral blood was collected every 2–4 weeks, to 14 weeks. Mice were euthanized and bone marrow and thymus removed at 8–14 weeks. For transgene detection in engrafted cells, each lineage of cells was FACS-sorted from bone marrow. Myeloid cells: CD33 APC (P67.6; BD), CD45 PE-Cy5 (J33; Coulter). B cells: CD19 PE (HIB19; BD), CD45 PE-Cy5 (J33; Coulter). T cells: CD3 PE-Cy7 (SK7; BD), CD45 PE-Cy5 (J33; Coulter). Between 10,000 and 50,000 cells were isolated with 2 or 3 biological replicates for multiple cell lines (iPSCs and ESCs). The QIAamp DNA Micro kit (Qiagen) was used to collect and prepare total genomic DNA for PCR detection of transgenes. Nested PCR reaction was performed similarly to the *in vitro* screening described in the above section.

### Mouse transplantation

NOD/LtSz-scidIL2Rgnull (NSG) mice (The Jackson Laboratory) were bred and housed at the Boston Children's Hospital animal care facility. Animal experiments were performed in accordance with institutional guidelines approved by Boston Children's Hospital Animal Care Committee. Intrafemoral transplantations were conducted with 6- to 10-week-old female mice irradiated (250 rad) 12 h before transplantation. Before transplantation, mice were temporarily sedated with isoflurane. A 26-half-gauge needle was used to drill the femur and a 0.3 × 10^5^ to 3.0 × 10^5^ range of cells was transplanted in a 25 μl volume using a 28.5-gauge insulin needle. Sulfatrim was administered in drinking water to prevent infections after irradiation. Doxycycline Rodent Diet (Envigo-Teklad Diets; 625 p.p.m.) and doxycycline (1.0 mg ml^−1^) were added to the drinking water to maintain transgene expression *in vivo* for 2 weeks (ref. 2). Secondary transplantation was performed with 1,000–3,000 human CD34^+^ cells (isolated from bone marrow by magnetic cell isolation with CD34 microbeads) at 8 weeks. Isolated cells were resuspended at 1,000–3,000 cells per 25 μl buffer (PBS + 2% FBS from StemCell Technologies) and kept on ice until injection. Cells were intrafemorally injected in to NSG mice. Sorted CD34^+^CD43^+^CD45^+^ (25,000 cells) or CD34^+^CD43^−^CD45^−^ (25,000 cells) HE-7TF cells were either intrafemorally or intravenously injected. For non-irradiated *c-Kit*-deficient immune-deficient recipients, the *NOD.Cg-Kit^W-41J^ Tyr*^+^ *Prkdc^scid^ Il2rg^tm1Wj^l*/ThomJ model was used (The Jackson Laboratory). Investigators were blinded for the analysis of mice. The experiments were not randomized. No statistical methods were used to predetermine sample size. For this analysis, multi-lineage engraftment was defined as chimaerism of human CD45^+^ cells in bone marrow encompassing four distinct lineages (myeloid, erythroid, B- and T-lymphoid, each comprising more than 1% of engrafted human CD45^+^ cells) in this study.

### Flow cytometry

Cells grown in EHT culture or harvested animal tissues were stained with the following antibody panels. Haemogenic endothelium panel: CD34 PE-Cy7 (8G12; BD), FLK1 AF647 (89106; BD), CD235a/glycophorin (GLY)-A FITC (11E4B-7-6; Coulter), and CD43 PE (1G10; BD). HSPC panel: CD38 PE-Cy5 (LS198-4-3; Clontech), CD34 PE-Cy7, and CD45 PE (HI30; BD). Lineage panel: CD235a/glycophorin (GLY)-A PE-Cy7 or FITC (11E4B-7-6; Coulter), CD33 APC (P67.6; BD), CD19 PE (HIB19; BD), IgM BV510 (G20-127; BD), CD4 PE-Cy5 (13B8.2; Coulter), CD3 PE-Cy7 (SK7; BD), CD8 V450 (RPA-T8; BD), TCRαβ BV510 (T10B9; BD), TCRγδ APC (B1; BD), CD45 PE-Cy5 (J33; Coulter), CD15 APC (HI98; BD), and CD31/PECAM PE (WM59; BD). All stains were performed with fewer than 1 × 10^6^ cells per 100 μl staining buffer (PBS + 2% FBS) with 1:100 dilution of each antibody, for 30 min at 4 °C in the dark. Compensation was performed by automated compensation with anti-mouse Igk negative beads (BD) and cord blood MNC stained with individual antibodies. All acquisitions were performed on a BD Fortessa cytometer. For detection of engraftment, human cord-blood-engrafted mouse marrow was used as a control to set gating; sorting was performed on a BD FACS Aria II cell sorter.

### Cytospin of erythroid cells and neutrophils

Five thousand to 10,000 FACS-sorted erythroid cells (CD235a/glycophorin (GLY)-A PE-Cy7 or FITC (11E4B-7-6; Coulter)), plasmacytoid lymphocytes (CD19 PE (HIB19; BD), IgM BV510 (G20-127; BD), CD38 PE-Cy5 (LS198-4-3; Clontech)), neutrophils (CD15 APC (HI98; BD), CD31/PECAM PE (WM59; BD) and CD45 PE-Cy5 (J33; Coulter)) were cytospun onto slides (500 r.p.m. for 10 min), air dried, and stained with May-Grunwald and Giemsa stains (both from Sigma), washed with water, air dried, and mounted, followed by examination by light microscopy.

### Quantitative PCR

RNA extraction was performed using an RNAeasy Microkit (Qiagen). Reverse transcription was performed using Superscript III (> 5,000 cells) or VILO reagent (< 5,000 cells) (Invitrogen). Quantitative PCR was performed in triplicate with SYBR Green (Applied Biosystems). Transcript abundance was calculated using the standard curve method. Primers used for globin genes were as follows^52^: huHbB F (5′-CTG AGG AGA AGT CTG CCG TTA-3′), huHbB R (5′-AGC ATC AGG AGT GGA CAG AT-3′), huHbG F (5′-TGG ATG ATC TCA AGG GCA C-3′), huHbG R (5′-TCA GTG GTA TCT GGA GGA CA-3′), huHbE F (5′-GCA AGA AGG TGC TGA CTT CC-3′), and huHbE R (5′-ACC ATC ACG TTA CCC AGG AG-3′).

### ELISA of terminally differentiated cells

FACS-isolated neutrophils (CD15^+^PECAM^+^CD45^+^) and T cells (CD3^+^CD45^+^) were cultured in IMDM + 10%FBS overnight in 96-well plates (flat-bottom), seeding 5,000–20,000 cells per well obtained from mice engrafted over 10% in primary recipients, or pooled mice engrafted less than 5% in primary recipients. Then supernatant was taken and analysed by MPO- or IFN-γ-ELISA –Ready-SET-Go! Kit (eBioscience) according to the manufacturer's protocol. The amount of IFN-γ was normalized per 1,000 cells. PMA (20 ng ml^−1^) and ionomycin (1 μg ml^−1^) were added to either neutrophils or T cells, then cells were cultured overnight (6–18 h). Human Ig production was measured from 50 μl of serum from NSG mice at 8 weeks (IgM) and 14 weeks after engraftment (IgG). Immunization of mice was done with OVA (F5503, Sigma) with Freund's complete adjuvant (F5881, Sigma), followed by booster doses of Freund's incomplete adjuvant (F5506, Sigma) according to the manufacturer's instructions. Six to 14 weeks after engraftment, mice were injected with antigen OVA (0.1%) emulsified in complete adjuvant subcutaneously at two sites on the back, injecting 100 μl at each site. A booster injection of antigen OVA (0.1%) emulsified in incomplete adjuvant was administered 14 days after immunization. The booster was given as a single subcutaneous injection with 100 μl at one site on the back. A serum sample was isolated from mice 7 days after the first booster dose, and human ova-specific antibody concentration was tested with an ovalbumin-specific IgG, OVA sIgG, ELISA Kit (Mybiosource, MB S700766) for human Ova-specific IgG and a Human Anti-Ovalbumin (Gal d 2) IgM ELISA Kit (Alpha Diagnostic, 670-145-OVM) to detect human Ova-specific IgG and IgM, respectively. The technical replicates were done with three measurements of the same experimental setup.

### T-cell receptor CDR3 sequencing

Human CD3^+^ T cells were FACS-isolated from thymus of engrafted NSG mice. Purified DNA was subjected to next-generation sequencing of CDR3 using immunoSEQ (Adaptive Biotechnology, Seattle, Washington, USA) and analysed with immunoSEQ Analyzer software (Adaptive Biotechnology).

### Affymetrix SNP 6.0 genotyping of engrafted cells

Aliquots (250 ng) of genomic DNA from human CD45^+^ bone marrow cells (CD45 PE-Cy5 (J33; Coulter)) from engrafted NSG mice and original PSCs (two biological replicates) were digested with either Nsp1 or Sty1. A universal adaptor oligonucleotide was then ligated to the digested DNAs. The ligated DNAs were diluted with water and three 10 μl aliquots from each well of the Sty 1 plate and four 10 μl aliquots from each well of the Nsp 1 plate were transferred to fresh 96-well plates. PCR master mix was added to each well and the reactions cycled as follows: 94 °C for 3 min; 30 cycles of 94 °C for 30 s, 60 °C for 45 s, 68 °C for 15 s; 68 °C for 7 min; 4 °C hold. After PCR, the seven reactions for each sample were combined and purified by precipitation from 2-propanol/7.5 M ammonium acetate. The ultraviolet absorbance of the purified PCR products was measured to ensure a yield ≥4 μg μl^−1^. Forty-five microlitres (≥ 180 μg) of each PCR product were fragmented with DNase 1 so the largest fragments were < 185 base pairs. The fragmented PCR products were then end-labelled with a biotinylated nucleotide using terminal deoxynucleotidyl transferase. For hybridization, the end-labelled PCR products were combined with hybridization cocktail, denatured at 95 °C for 10 min and incubated at 49 °C. Two hundred microlitres of each mixture was loaded on a GeneChip and hybridized overnight at 50 °C and 60 r.p.m. After 16–18 h of hybridization, the chips were washed and stained using the GenomeWideSNP6_450 fluidics protocol with the appropriate buffers and stains. After washing and staining, the GeneChips were scanned on a GeneChip Scanner 3000 using AGCC software. Genotype calls (chp files) were generated in Affymetrix Genotyping Console using the default parameters. The resulting chp files were analysed for familial relationships using the identity by state algorithm implemented in Partek Genomics Suite.

### RNA-seq

Engrafted human CD34^+^CD38^−^CD45^+^ HSCs were isolated from bone marrow from either iPS-derived haemogenic endothelium- or cord-blood-injected NSG mice, then RNA was purified with an RNeasy Micro kit (Qiagen). Quality control of RNA was done by Bioanalyzer and qubit analysis. Samples that passed quality control were converted into libraries and sequenced by a Nextseq PE75 kit. Raw reads were aligned to the human genome/transcriptome using TopHat2 software^53^. Gene expression levels and reads per kilobase per million (RPKM) values were estimated using a htseq-count tool^54^ and the edgeR package^55^. For a legitimate transcriptome-wide comparison, we retrieved raw RNA-seq data of two published from the Gene Expression Omnibus database (Long non-coding RNA profiling of human lymphoid progenitors reveals transcriptional divergence of B-cell and T-cell lineages, accession number GSE69239; Distinct routes of lineage development reshape the human blood hierarchy across ontogeny, accession number GSE76234) and calculated RPKM values using a same analysis pipeline.

### Single-cell RNA-seq with in-droplet-seq technology

Engrafted human CD34^+^CD38^−^CD45^+^ HSCs were isolated from bone marrow from either iPS-derived haemogenic endothelium- or cord-blood-injected NSG mice, then processed for in-droplet barcoding according to a previous report^29^. The library was QCed with Bioanalyzer and sequenced by Nextseq PE 75 kit. The t-SNE algorithm was used to visualize transcriptome similarities and population heterogeneity of cord blood HSCs and iPSC-derived HSCs. The t-SNE algorithm performs a dimensionality reduction of multidimensional single-cell RNA-seq data into a low-dimensional space, preserving pairwise distances between data points as well as possible, allowing a global visualization of subpopulation structure and cell–cell similarities. We used the R package tsne in our analyses. The t-SNE map was initialized with point-to-point distances computed by classical multidimensional scaling, and the R plot function was used to visualize t-SNE maps annotated by cord blood or iPSC-derived HSCs. Plots showing t-SNE maps coloured by expression of selected genes were created using the ggplot2 package. For subpopulation identification, we used the top 500 genes with the highest variance to elucidate global differences among single cells. To assess transcriptome similarities in terms of induction of haematopoietic genes in iPSC-derived HSCs, we used 62 haematopoietic genes for t-SNE analysis in Supplementary Table 2.

### Gene set enrichment analysis

Gene set enrichment analysis was performed with the desktop client version (javaGSEA, http://software.broadinstitute.org/gsea/downloads.jsp) with default parameters. RPKM values from the 7F-HSPC were obtained from the RNA-seq (described previously). These values were normalized to a terminally differentiated cell and the normalized values were used to rank the most differentially expressed genes. These differentially expressed genes were used to run gene set enrichment analysis with gene sets obtained from mSigDB (KEGG, Hallmark, immunological, transcription factors, and chemical and genetic perturbations gene sets were used). In addition, gene sets specific to progenitors, cord blood, or fetal-liver HSC were obtaine d from previous reports^16,56^. FDR < 0.25 with *P* < 0.05 was considered significant.

### Lentiviral integration detection by ligation-mediated PCR and next-generation sequencing

CD33^+^ myeloid cells, CD19^+^ B cells, and CD3^+^ T cells were isolated from bone marrow from haemogenic-endothelium-injected NSG mice. Genomic DNA was purified with a QIAamp DNA Micro kit (Qiagen). Ligation-mediated PCR-based detection of lentiviral integration sites was done with a Lenti-X Integration Site Analysis Kit (Clontech) according to the manufacturer's instructions. Sequencing-based detection (integration sequencing) was done as previously described^57^.

### Data availability

RNA-seq from this study have been deposited in the Gene Expression Omnibus under accession number GSE85112. We retrieved raw RNA-seq data of two published from the Gene Expression Omnibus database (Long non-coding RNA profiling of human lymphoid progenitors reveals transcriptional divergence of B-cell and T-cell lineages, accession number GSE69239; Distinct routes of lineage development reshape the human blood hierarchy across ontogeny, accession number GSE76234). The data are all in the paper, or are available from the corresponding author upon reasonable request if not.

## Extended Data

## Supplementary Material

Supplemental
