# Supplementary material for: Haematopoietic stem and progenitor cells from human pluripotent stem cells
Source: Nature. Author manuscript; Available in PMC 2018 Mar 28. (PMC5872146; doi:10.1038/nature22370)
Supplement: Supplemental [file NIHMS898882-supplement-Supplemental.pdf]

doi:10.1038/nature22370

Uncropped gel data

ED 4c iPSC -1

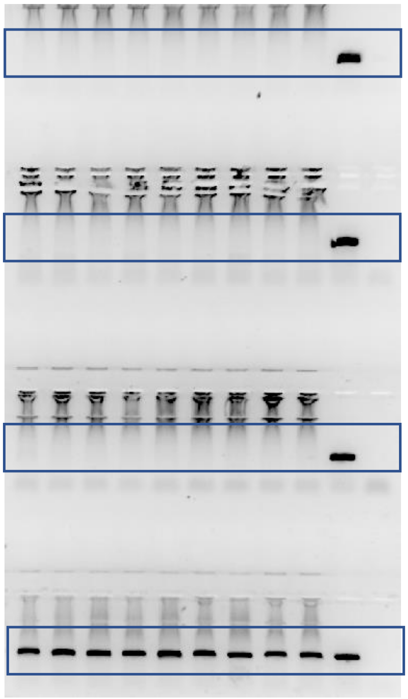

EVI1

MAFF

KLF4

SPI1

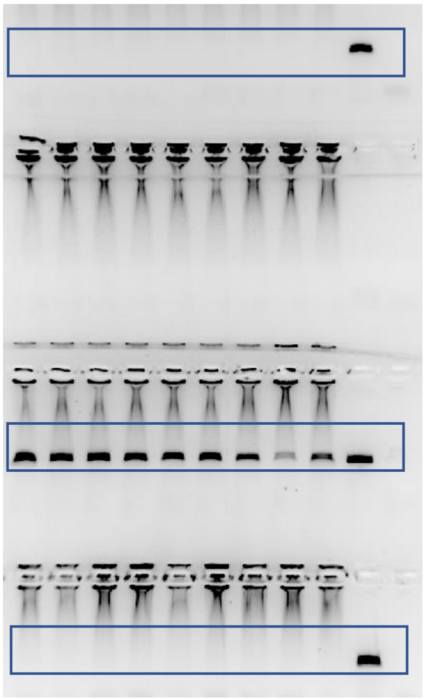

FOSB

ERG

SOX4

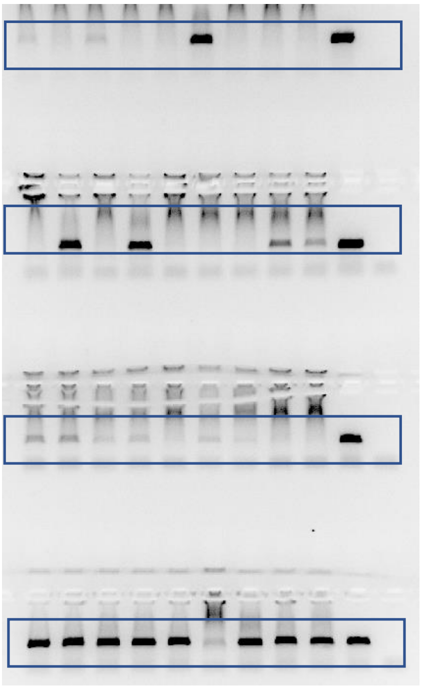

HLF

NRIP1

IRF2

HOXA10

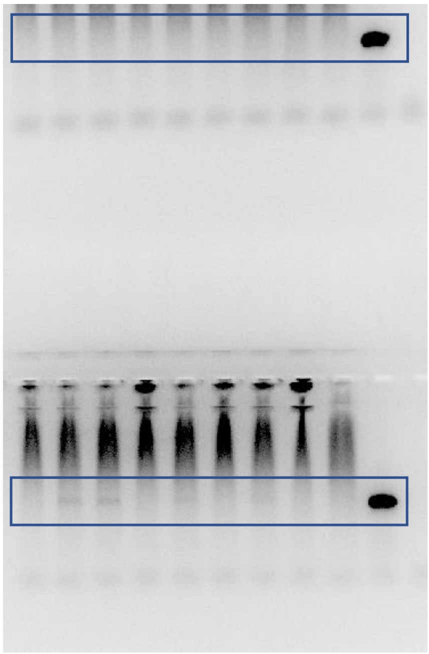

MYB

BCL11A

Uncropped gel data

ED 4c iPSC -2

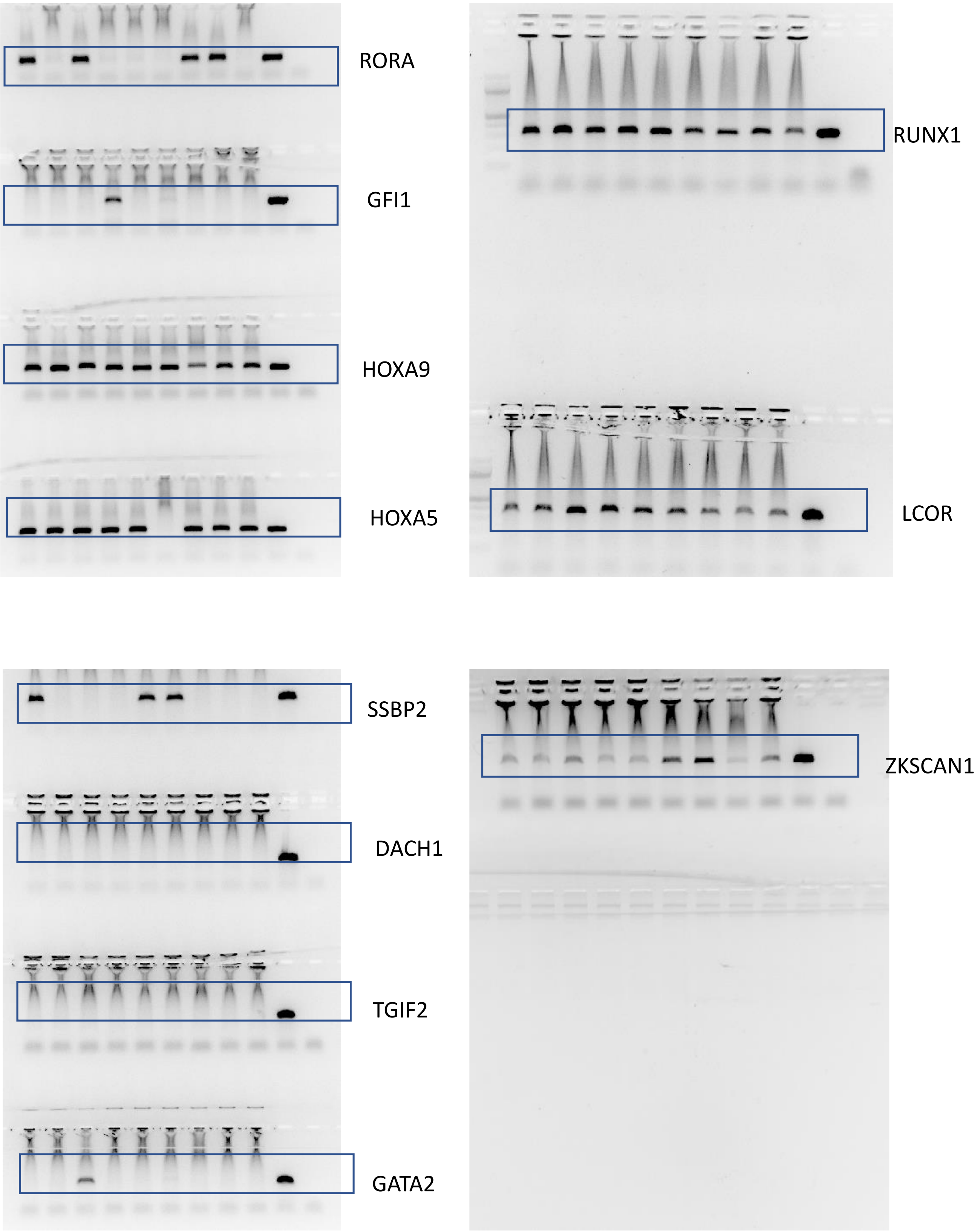

Uncropped gel data

ED 4c hESC -1

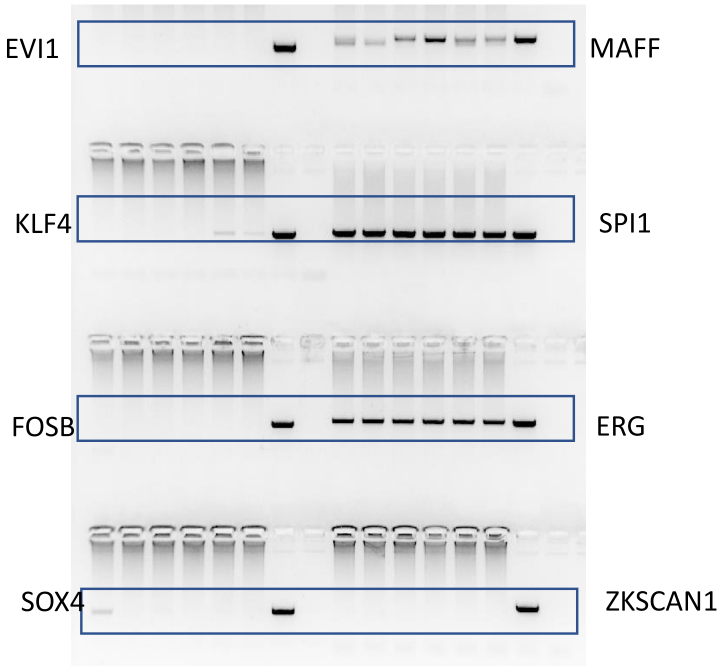

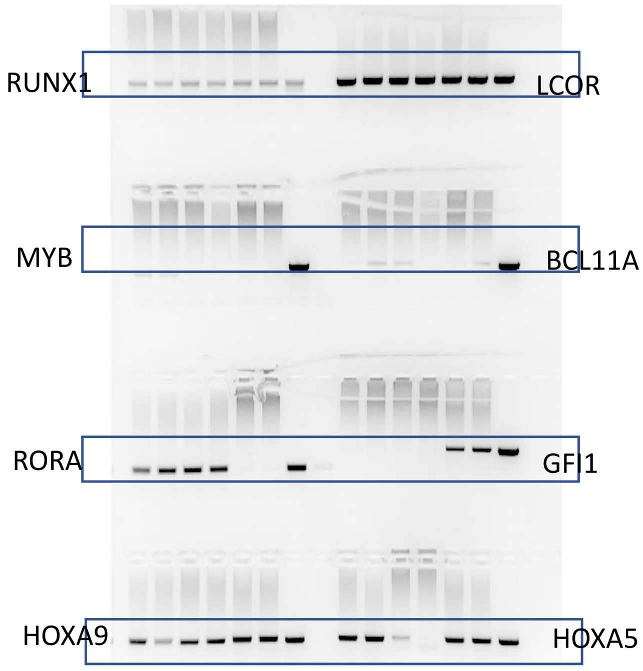

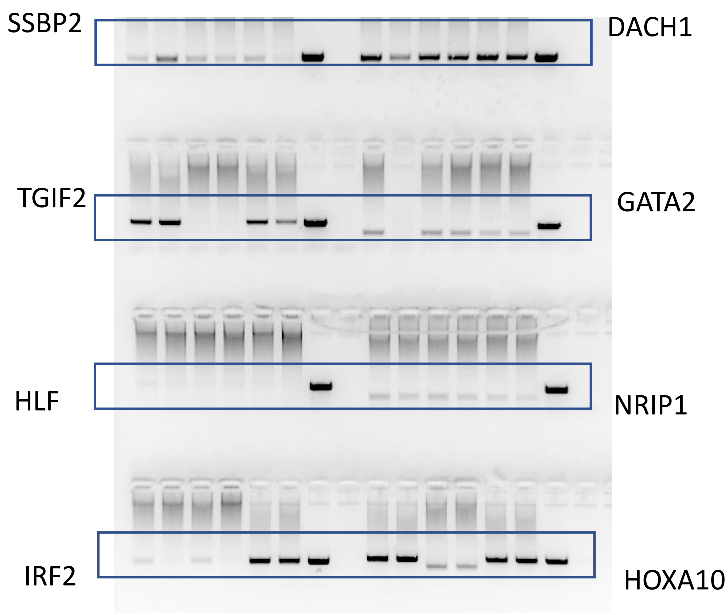

Uncropped gel data

ED 4c hESC -2

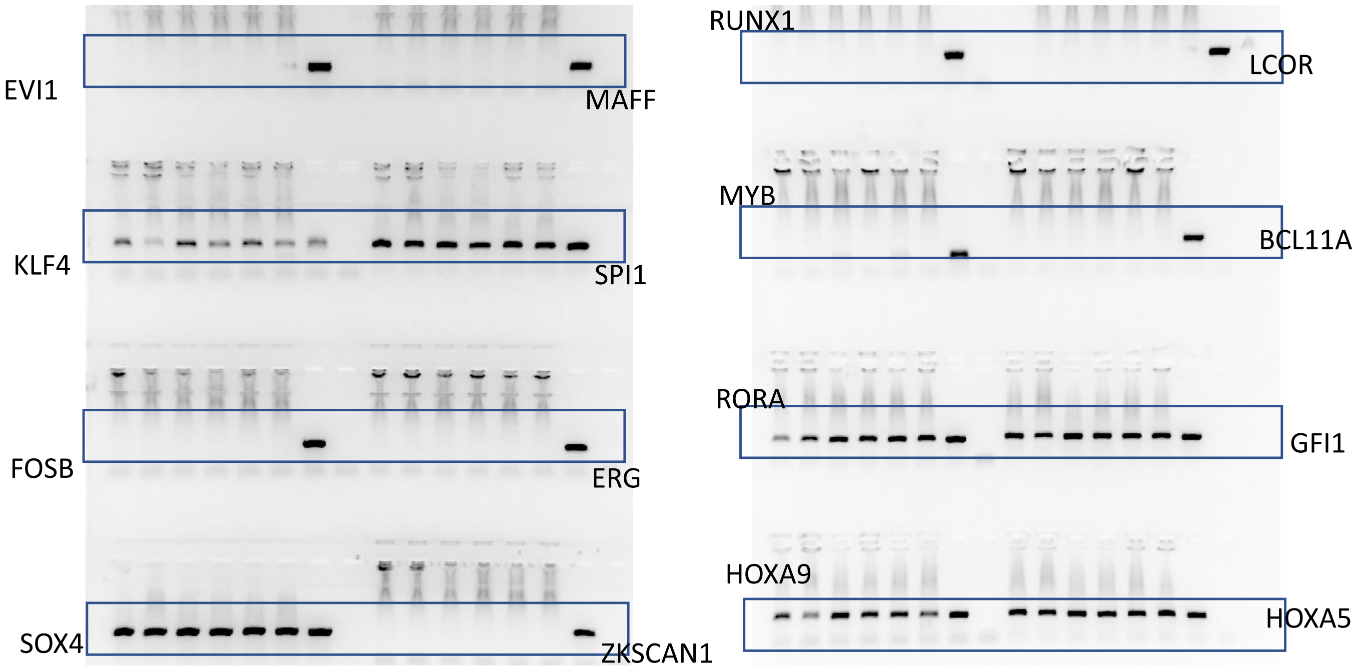

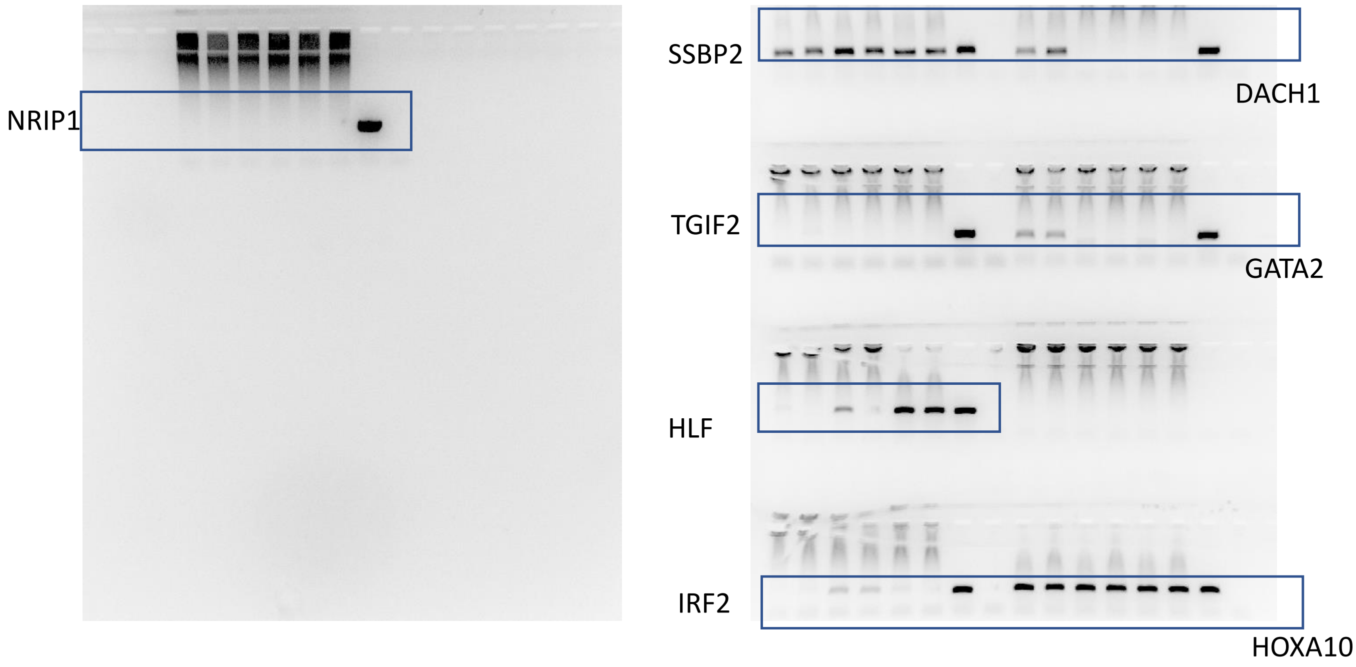

Uncropped gel data

ED 5e

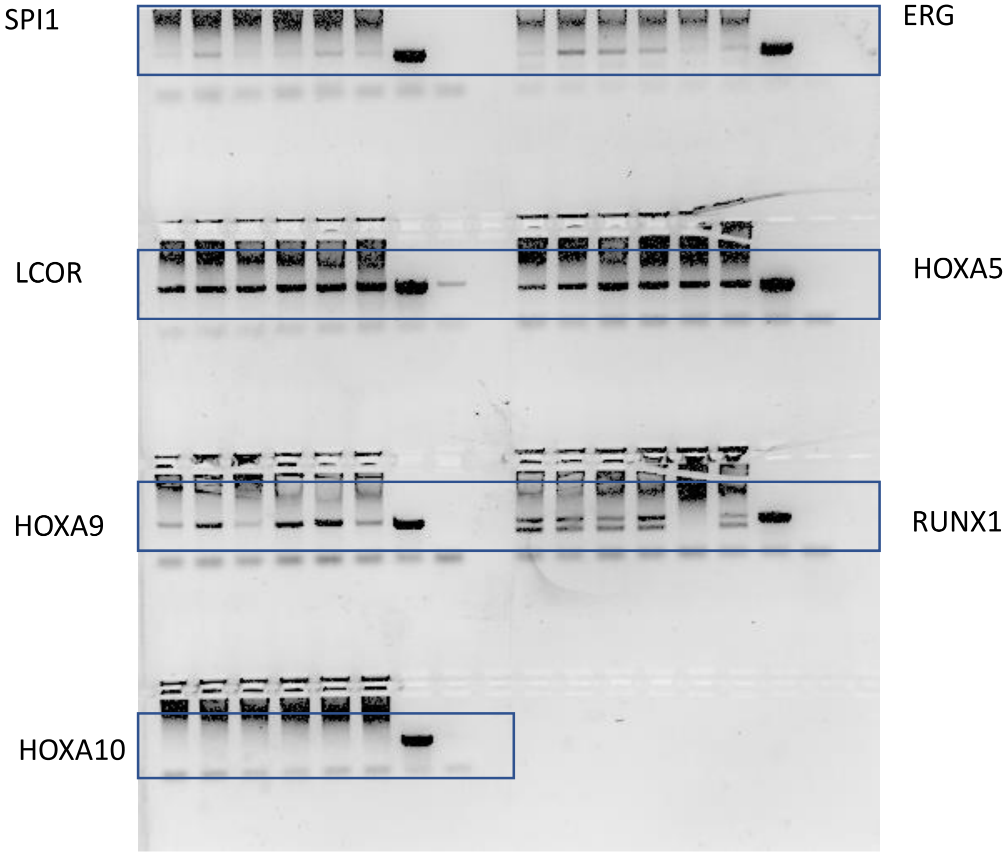

Comparison with CB-HSCs

	7TF HSPC	CB-HSC
Sustainability	Secondary - 14 weeks	Tertiary
Multilineage	Yes, but CD8+ T cells	Yes
Multipotent clonality	Yes	Yes
Cell cycle	Fast	Slow
Robustness of engraftment	20-30% vs CB	
Functionality of mature cells	30-60% vs CB	
Expression level of HSC signature	30-80% vs CB	
Molecular features	Partial lineage program upon engraft	Full lineage program upon engraft

Table 1
Comparison of functional and molecular properties of HE-7TF cells and CB-HSCs.
Features of HE-7TF cells compared with CB-HSCs. The table summarizing the difference between HE-7TF cells and CB-HSCs. Consistent with molecular-level profiling, HE-7TF cells mediate shorter term engraftment and lower functionality than CB-HSCs, indicating potential opportunities for improvement.

1	ENSG00000173585
2	ENSG00000004468
3	ENSG00000012124
4	ENSG00000182866
5	ENSG00000245848
6	ENSG00000148400
7	ENSG00000081059
8	ENSG00000275122
9	ENSG00000115085
10	ENSG00000128218
11	ENSG00000164105
12	ENSG00000106004
13	ENSG00000174059
14	ENSG00000164687
15	ENSG00000153563
16	ENSG00000106006
17	ENSG00000164330
18	ENSG00000177455
19	ENSG00000066336
20	ENSG00000196549
21	ENSG00000126756
22	ENSG00000107485
23	ENSG00000172005
24	ENSG00000173762
25	ENSG00000182742
26	ENSG00000157404
27	ENSG00000127152
28	ENSG00000172673
29	ENSG00000107447
30	ENSG00000116824
31	ENSG00000010610
32	ENSG00000078399
33	ENSG00000161405
34	ENSG00000165702
35	ENSG00000143995
36	ENSG00000135363
37	ENSG00000188846
38	ENSG00000198851
39	ENSG00000119866
40	ENSG00000253293
41	ENSG00000169575
42	ENSG00000007062
43	ENSG00000122592
44	ENSG00000179348
45	ENSG00000162676
46	ENSG00000117400
47	ENSG00000118513
48	ENSG00000104903
49	ENSG00000122025
50	ENSG00000171611
51	ENSG00000166349
52	ENSG00000168685
53	ENSG00000157554
54	ENSG00000102145
55	ENSG00000134954
56	ENSG00000196092
57	ENSG00000111275
58	ENSG00000162367
59	ENSG00000158477
60	ENSG00000159216
61	ENSG00000126353
62	ENSG00000084774
